# Predicting the accuracy of genomic predictions

**DOI:** 10.1186/s12711-021-00647-w

**Published:** 2021-06-29

**Authors:** Jack C. M. Dekkers, Hailin Su, Jian Cheng

**Affiliations:** Department of Animal Science, Iowa State University, Ames, Iowa, USA

## Abstract

**Background:**

Mathematical models are needed for the design of breeding programs using genomic prediction. While deterministic models for selection on pedigree-based estimates of breeding values (PEBV) are available, these have not been fully developed for genomic selection, with a key missing component being the accuracy of genomic EBV (GEBV) of selection candidates. Here, a deterministic method was developed to predict this accuracy within a closed breeding population based on the accuracy of GEBV and PEBV in the reference population and the distance of selection candidates from their closest ancestors in the reference population.

**Methods:**

The accuracy of GEBV was modeled as a combination of the accuracy of PEBV and of EBV based on genomic relationships deviated from pedigree (DEBV). Loss of the accuracy of DEBV from the reference to the target population was modeled based on the effective number of independent chromosome segments in the reference population (*M*_*e*_). Measures of *M*_*e*_ derived from the inverse of the variance of relationships and from the accuracies of GEBV and PEBV in the reference population, derived using either a Fisher information or a selection index approach, were compared by simulation.

**Results:**

Using simulation, both the Fisher and the selection index approach correctly predicted accuracy in the target population over time, both with and without selection. The index approach, however, resulted in estimates of *M*_*e*_ that were less affected by heritability, reference size, and selection, and which are, therefore, more appropriate as a population parameter. The variance of relationships underpredicted *M*_*e*_ and was greatly affected by selection. A leave-one-out cross-validation approach was proposed to estimate required accuracies of EBV in the reference population. Aspects of the methods were validated using real data.

**Conclusions:**

A deterministic method was developed to predict the accuracy of GEBV in selection candidates in a closed breeding population. The population parameter *M*_*e*_ that is required for these predictions can be derived from an available reference data set, and applied to other reference data sets and traits for that population. This method can be used to evaluate the benefit of genomic prediction and to optimize genomic selection breeding programs.

**Supplementary Information:**

The online version contains supplementary material available at 10.1186/s12711-021-00647-w.

## Background

As was proposed by Meuwissen et al. [1], genomic selection involves the use of genotypes from high-density single nucleotide polymorphisms (SNPs) to estimate so-called genome-enhanced or genomic estimated breeding values (GEBV) based on genomic prediction. Genomic prediction requires a training or reference population of individuals that have been genotyped and phenotyped in order to predict the GEBV of individuals in the target population, i.e. the selection candidates, based on their SNP genotypes without requiring them to be phenotyped. Genomic selection promises to increase rates of genetic improvement by enabling higher accuracy of EBV at a young age. While the original concept of genomic prediction was based on SNPs that capture population-wide linkage disequilibrium (LD) between markers and quantitative trait loci (QTL), Habier et al. [2] showed that, in a closed population, pedigree information and co-segregation between QTL and SNPs can also substantially contribute to the accuracy of GEBV.

The design of breeding programs with genomic selection requires methods to *a-priori* predict the accuracy of GEBV. During the past decade, several deterministic approaches have been developed for this purpose based on population parameters. Dekkers [3] showed that the accuracy of GEBV is the product of the square root of the proportion of genetic variance that is captured by the SNP panel ($q$) and the accuracy with which genetic effects that are captured by the SNPs can be estimated ($r$). Daetwyler et al. [4] showed that the latter depends on the heritability of the phenotypes in the reference dataset, the size of the reference population, and the number of independent QTL that affect the trait. These concepts were further developed by Goddard [5], Daetwyler et al. [6], Hayes et al. [7], Goddard et al. [8], Meuwissen [9], Erbe et al. [10], Wientjes et al. [11], and others. A key population parameter in these predictions is the effective number of chromosome segments, ${M}_{e},$ which was first introduced by Visscher et al. [12] to represent the number of independent genetic (QTL) effects that are estimated based on the available SNP genotypes. Visscher et al. [12] and Goddard [5] showed that ${M}_{e}$ can be derived based on historical effective population size and size of the genome. Based on these concepts, multiple deterministic formulae have been developed to predict ${M}_{e}$ [5, 8, 13–15]. Brard and Ricard [16] compared several of these and found that they result in very different estimates of ${M}_{e}$ and, therefore, in very different accuracies of GEBV, with none providing accurate predictions across a range of programs.

As an alternative, Hayes et al. [7] and Goddard et al. [8] showed that ${M}_{e}$ within a population is equal to the reciprocal of the variance of deviations of genomic relationships from their pedigree-based expectations, while Wientjes et al. [11] showed that, across populations, ${M}_{e}$ can be estimated from a genomic relationship matrix that combines both populations. Thus, if a sufficient number of individuals within a population is genotyped, an empirical estimate of ${M}_{e}$ can be derived from the population’s genomic and pedigree-based relationship matrices. However, recently, van den Berg et al. [17] showed that the use of ${M}_{e}$ derived from the variance of relationships results in overestimates of the accuracy of GEBV in dairy cattle populations.

To address the inadequacy of theoretical predictions of GEBV, Erbe et al. [10] derived empirical adjustments to the deterministic predictions of accuracy of [4] based on observed accuracies from cross-validation. Brard and Ricard [16] also proposed to derive ${M}_{e}$ empirically from the observed accuracy of GEBV in the population, as originally proposed by Daetwyler et al. [6]. A similar conclusion was recently reached by van den Berg et al. [17], who showed that a parameter that is related to ${M}_{e}$ can be estimated from a reference dataset for a population and used as a population parameter to predict accuracies of GEBV obtained from other reference datasets from that population, including for phenotypes with different heritabilities. Thus, until a better theoretical foundation is obtained, deriving an empirical estimate of ${M}_{e}$ from a relevant reference dataset as a population parameter appears to be the only solution to obtain the required parameters to predict accuracies of GEBV for different reference datasets for that population. Although this limits applications to breeding programs that already have a reference population, it should be noted that ongoing genomic selection programs require many decisions to be evaluated and optimized, including which animals to genotype and which animals to phenotype for which traits and at what age. All these decisions require the ability to model the accuracy of GEBV.

A key controversy in the development of methods to predict the accuracy of GEBV has been whether ${M}_{e}$ should be derived based on the reference population or based on the relationship of the reference to the target population, or both. Several studies have shown that the accuracy of GEBV declines as the distance between the reference and target population increases [2, 18]. Habier et al. [2] showed that this decline in accuracy is the result of the break-up of LD between SNPs and QTL between the reference and target populations and of the decline in pedigree relationships and pedigree information that is implicit to GEBV. Goddard et al. [8] suggested that ${M}_{e}$ should be based on the variance of relationships between the reference and target populations and this has been applied by several [11, 17]. However, the accuracy with which the effect of chromosomal segments can be estimated should depend on ${M}_{e}$ in the reference population, not on ${M}_{e}$ between the reference and target populations, although the latter may affect the loss in accuracy between the reference and target populations. Clark et al. [19] showed that the accuracy of GEBV of selection candidates depends on their maximum relationship with individuals in the reference population, rather than on the variance of those relationships. Similarly, Pszczola et al. [20] showed that the accuracy of genomic predictions for a target population can be maximized by minimizing relationships within the reference population and by maximizing relationships between the reference and target populations. For across-population genomic prediction, Wientjes et al. [21] showed that the consistency of marker-QTL LD between the reference and target populations is an important factor to explain the much lower accuracy of GEBV in across-population versus within-population prediction. Wientjes et al. [21] quantified this consistency based on the accuracy with which a selection index that was derived to predict QTL genotype based on SNP genotypes within the reference population, can predict QTL genotypes in the target population.

Habier et al. [2, 22] showed that it is important to differentiate between contributions of pedigree, co-segregation, and LD information to GEBV when investigating and modeling the accuracy of GEBV because each of these accumulates and erodes at a different rate. However, this has not been explicitly considered when deriving deterministic predictions of the accuracy of GEBV, with some exceptions. For example, van den Berg et al. [17] used the concept of Fisher’s information to adjust the accuracy of GEBV for the contribution of pedigree information when combining genomic information from two related reference populations.

Deterministic methods to predict response to selection are important for the design and optimization of breeding programs. Within the context of genomic selection, such methods must be able to account for the contribution of pedigree versus genomic information and for the relationship of selection candidates with animals in the training population in order to compare breeding programs that differ in which and when animals are genotyped and phenotyped relative to when selection decisions are made. Methods to model the accuracy of GEBV that take these aspects into account have not been developed.

Against this background, the objectives of this study were to: (1) develop a deterministic approach to model and predict the accuracy of GEBV for selection candidates in a closed breeding population by explicitly modeling the contribution of pedigree versus genomic information and the relationship between the reference and target populations, and (2) develop an empirical estimate of ${M}_{e}$ based on a reference population, that can be used as a population parameter in the above predictions for use across reference dataset sizes and traits for that population. Although this limits applications to situations where a reference population is available, this is now the case for most ongoing breeding programs. The developed method will enable further optimization of genomic selection breeding programs, including determining which animals should be genotyped and which should be phenotyped for which traits and at what age, among others. Simulation will be used to demonstrate that the developed methods result in accurate predictions of the accuracy of GEBV within a closed population, both without and with selection, while real data will be used to test some of the assumptions made.

## Methods

### General modelling strategy

The trait considered is assumed to follow the pseudo-infinitesimal additive model, i.e. phenotype is affected by many additive QTL with small effects across the genome, as well as by random environmental effects. In the following, predictions of breeding values are assumed to be based on best linear unbiased prediction (BLUP) [23], using either pedigree or genomic relationships. Following Legarra and Ducrocq [24], GEBV can be partitioned into a part that can be captured by pedigree relationships and a part that can be captured by genomic relationships deviated from pedigree relationships:

${\widehat{g}}_{A}$ = EBV based on pedigree relationships (PEBV), with accuracy ${r}_{A},$

${\widehat{g}}_{D}$ = EBV based on genomic deviated from pedigree relationships (DEBV), with accuracy ${r}_{D},$

${\widehat{g}}_{G}$ = EBV based on genomic relationships (GEBV), with accuracy ${r}_{G}.$

Following Legarra and Ducrocq [24], ${\widehat{g}}_{D}$ could be obtained from a model in which the breeding value, ${\mathbf{g}}_{\boldsymbol{G}}$ is partitioned into a pedigree-based component, ${\mathbf{g}}_{\boldsymbol{A}},$ and a genomic minus pedigree component, ${\mathbf{g}}_{\boldsymbol{D}},$ as: ${\mathbf{g}}_{\boldsymbol{G}}={\mathbf{g}}_{\boldsymbol{A}}+{\mathbf{g}}_{\boldsymbol{D}},$ with variance–covariance matrix: $$Var\left[ {\begin{array}{*{20}{c}} {{{\mathbf{g}}_G}} \\ {{{\mathbf{g}}_A}} \\ {{{\mathbf{g}}_D}} \end{array}} \right] = \left[ {\begin{array}{*{20}{c}} {\mathbf{G}}&{\mathbf{A}}&{{\mathbf{G}} - {\mathbf{A}}} \\ {\mathbf{A}}&{\mathbf{A}}&{\mathbf{0}} \\ {{\mathbf{G}} - {\mathbf{A}}}&{\mathbf{0}}&{{\mathbf{G}} - {\mathbf{A}}} \end{array}} \right]\sigma _g^2,$$ where $\mathbf{A}$ and $\mathbf{G}$ are the pedigree-based and genomic relationship matrices, respectively, and ${\sigma }_{g}^{2}$ is the genetic variance. Legarra and Ducrocq [24] showed that this model is equivalent to the standard GBLUP model that fits ${\mathbf{g}}_{\boldsymbol{G}}$ with the genomic relationship matrix $\mathbf{G}.$ In the approach that will be used here, however, ${\widehat{g}}_{A}$ represents EBV from standard pedigree BLUP, using only the pedigree-based relationship matrix $\mathbf{A},$ while ${\widehat{g}}_{D}$ represents EBV based on ($\mathbf{G}-\mathbf{A})$ as relationship matrix. Note that ${\widehat{g}}_{D}$ exists only in concept, representing contributions of phenotypes to GEBV through deviations of genomic from pedigree relationships. This approach was used to model the contribution of pedigree versus genomics to GEBV, instead of the Legarra and Ducrocq [24] approach, because deterministic models have been well developed for the accuracy of conventional pedigree-based BLUP EBV ([25, 26]), but not for the pedigree-based EBV obtained from the Legarra and Ducrocq [24] model.

The three EBV, ${\widehat{g}}_{A},$ ${\widehat{g}}_{D},$ and ${\widehat{g}}_{G},$ can be defined for both the reference and the target population and their corresponding accuracies, ${r}_{A},$ ${r}_{D},$ and ${r}_{G},$ are related to each other, as will be described below. Throughout this paper, accuracies will refer to population accuracies, rather than individual accuracies, as defined by [27], because population accuracies are relevant for prediction of response to selection.

**Fig. 1:**
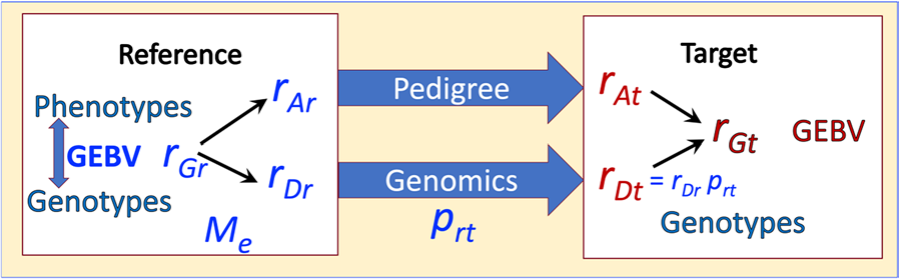
Illustration of the general strategy of predicting the accuracy of genomic estimated breeding values (GEBV) in the target population. The accuracy of GEBV in the target $(t)$ population is predicted based on the accuracy of GEBV in the reference $(r)$population of genotyped and phenotyped individuals, by separating the accuracy of GEBV (${r}_{G}$) into the accuracy of pedigree information (${r}_{A}$) and genomics (${r}_{D}$), using ${M}_{e}$ as a key parameter of the reference population and ${p}_{rt}$ representing the loss of accuracy of genomic information from the reference to the target population

The general strategy that will be used here to predict the accuracy of GEBV in the target population is illustrated in Fig. 1. The goal is to predict the accuracies of ${\widehat{g}}_{A}$ and ${\widehat{g}}_{D}$ in the target population, which are then combined to predict the accuracy of ${\widehat{g}}_{G}$ in the target population based on the relationship of ${r}_{A}$ and ${r}_{D}$ with ${r}_{G}.$ The accuracy of pedigree-based EBV, ${\widehat{g}}_{A},$ in the target population, ${r}_{{A}_{t}},$ can be derived using standard pseudoBLUP approaches [25, 26], e.g. as implemented in the software SelAction [28]. Although, typically, selection candidates will not have own phenotype in genomic selection programs, this is not required for the proposed approach, as own phenotype can be accommodated in the pedigree-based predictions. The accuracy of ${\widehat{g}}_{D}$ in the target population, ${r}_{{D}_{t}},$ depends on the accuracy of ${\widehat{g}}_{D}$ in the reference population, ${r}_{{D}_{r}},$ and the decline in accuracy from the reference to the target population, ${p}_{rt}.$ The accuracy of ${\widehat{g}}_{D}$ in the reference population could be derived using theoretical methods previously developed [4, 5, 17] based on size of the reference population, heritability, and ${M}_{e}$ in the reference population. However, given the limitations of these methods to estimate ${M}_{e},$ as described in the Background section, here, two empirical methods will be explored to derive ${M}_{e}$ in the reference population based on observed accuracies of ${\widehat{g}}_{A}$ and ${\widehat{g}}_{G}$ in that population. A pseudo code for the developed method is in Appendix 1.

### Basic predictive relationships

The accuracy of ${\widehat{g}}_{D}$ in the reference population can be derived based on the following relationship, after Daetwyler et al. [4] and van den Berg et al. [17]:

1 $${r}_{{D}_{r}}^{2}=\frac{{q}_{D}^{2}{\theta }_{{D}_{r}}}{1+{\theta }_{{D}_{r}}-{r}_{{D}_{r}}^{2}{q}_{D}^{2}{h}^{2}},$$

where ${h}^{2}$ is the heritability of the phenotypes used for training, ${q}_{D}^{2}$ is the proportion of genetic variance that is captured by genomics, and

2 $${\theta }_{{D}_{r}}=N{q}_{D}^{2}{h}^{2}/{M}_{e},$$

where $N$ is the size of the reference population and ${M}_{e}$ is the effective number of chromosome segments in the reference population, as defined by Visscher et al. [12], Goddard [5], and Hayes et al. [7]. Equations (1) and (2) are modified from van den Berg et al. [17] by accounting for ${q}^{2}<1,$ which enters Eqs. (1) and (2) in two ways: (i) ${q}^{2}$ affects the marker-based heritability of the phenotypes (= ${q}^{2}{h}^{2}$), which appears in the denominator of Eq. (1) and in the numerator of Eq. (2); (ii) ${q}^{2}$ reduces the squared accuracy of genomic information as a predictor of the breeding value [3], which enters ${q}^{2}$ in the numerator of Eq. (1). When the distribution of minor allele frequencies of markers is the same as that of QTL, Goddard [8] showed that ${q}^{2}$ can be derived as a function of the number of genotyped markers ($M$) and ${M}_{e}$ as:

3 $${q}_{D}^{2}=M/(M+{M}_{e}).$$

Note that for ${\widehat{g}}_{A}$ and ${\widehat{g}}_{G},$ ${q}_{A}^{2}={q}_{G}^{2}=1,$ as both ${\widehat{g}}_{A}$ and ${\widehat{g}}_{G}$ include pedigree information, which covers the entire genome. The general form of Eq. (1) can be solved for ${\theta }_{i}$ for each component *i* (= A, D, G) as:

4 $${\theta }_{i}=\frac{{r}_{i}^{2}(1-{r}_{i}^{2}{q}_{i}^{2}{h}^{2})}{{q}_{i}^{2}-{r}_{i}^{2}}.$$

### Relationship between $${\boldsymbol{r}}_{\boldsymbol{G}},$$$${\boldsymbol{r}}_{\boldsymbol{A}}$$ and $${\boldsymbol{r}}_{\boldsymbol{D}}$$

The relationship between accuracies ${\widehat{g}}_{G}{,\widehat{g}}_{A},$ and ${\widehat{g}}_{D}$ in either the reference or the target population was derived by two approaches: using Fisher’s information statistics (Fisher) and using selection index theory (Index). Both approaches are based on the assumption that the sampling errors of ${\widehat{g}}_{A}$ and ${\widehat{g}}_{D}$ are independent of each other, following van den Berg et al. [17].

#### Fisher information approach

Parameter ${\theta }_{D}$ in Eqs. (1) and (2) is proportional to $N$ and, as noted by van den Berg et al. [17], represents Fisher’s information of ${\widehat{g}}_{D},$ which quantifies the amount of information about the true breeding value that is contained in ${\widehat{g}}_{D}$ [29]. The general form of Eq. (1) can also be applied to the pedigree-based and genomic EBV, ${\widehat{g}}_{A}$ and ${\widehat{g}}_{G},$ with their corresponding Fisher’s information statistics, ${\theta }_{A}$ and ${\theta }_{G}$_._ Based on standard statistical theory [29], under the assumption of independence of sampling errors, the sum of the Fisher’s information statistics of ${\widehat{g}}_{A}$ and ${\widehat{g}}_{D}$ is equal to Fisher’s information of ${\widehat{g}}_{G}$ [17], i.e.:

5 $${\theta }_{G}={\theta }_{A}+{\theta }_{D}.$$

When estimates of ${r}_{G}$ and ${r}_{A}$ are available, Eq. (4) can be used to compute ${\theta }_{G}$ and ${\theta }_{A}$ after which ${\theta }_{D}$ can be computed as ${\theta }_{D}={\theta }_{G}-{\theta }_{A}$ based on Eq. (5).

Equation (4) can also be converted into the following quadratic form for ${r}_{i}^{2}:$

${{q}_{i}^{2}{h}^{2}r}_{i}^{4}+\left(-1-{\theta }_{i}\right){r}_{i}^{2}+{q}_{i}^{2}{\theta }_{i}=0,$ which can be solved for ${r}_{i}^{2}$ as:

6 $$r_i^2 = \left[ {1 + {\theta _i} - \sqrt {{{\left( {1 + {\theta _i}} \right)}^2} - 4{h^2}q_i^4{\theta _i}} } \right]/2q_i^2{h^2}.$$

With ${M}_{e}$ and, therefore, ${q}_{D}^{2}$ known (see later), Eq. (6) can then be used to compute ${r}_{D}.$

#### Selection index approach

The selection index to combine ${\widehat{g}}_{A}$ and ${\widehat{g}}_{D}$ is:

7 $${\widehat{g}}_{G}={b}_{A}{\widehat{g}}_{A}+{b}_{D}{\widehat{g}}_{D}.$$

Using standard selection index theory [30] and assuming that sampling errors of ${\widehat{g}}_{A}$ and ${\widehat{g}}_{D}$ are independent, the squared accuracy of this index can be derived to be (see Appendix 2):

8 $$r_G^2 = \frac{{r_A^2 + r_D^2 - 2r_A^2r_D^2}}{{1 - r_A^2r_D^2}},$$

which can be used to compute ${r}_{D}^{2}$ as:

9 $${r}_{D}^{2}=\frac{{r}_{G}^{2}{-r}_{A}^{2}}{{1+r}_{A}^{2}({r}_{G}^{2}-2)}.$$

Similar relationships were previously derived by Harris and Johnson [31], among others, to compute the accuracy of animal model EBV based on pedigree, own, and progeny data as sources of information with independent sampling errors. Note that the Fisher and Index approaches do not result in the same value for ${r}_{D}$ for given values of ${r}_{G}$ and ${r}_{A}$.

### ${M}_{e}$ for the reference population

If an estimate of ${M}_{e}$ for the reference population is available, the accuracy of GEBV in the reference population can be estimated using Eqs. (1), (2), and (3) to predict ${r}_{{D}_{r}}.$ Cross-validation or pseudoBLUP methodology can be used to predict ${r}_{{A}_{r}}.$ These two accuracies can then be combined to predict ${r}_{{G}_{r}},$ using either the Fisher approach (Eqs. (6) and (7)) or the Index approach (Eq. (9)).

If ${M}_{e}$ for the reference population is not known, it can be derived using different approaches:

- Based on theoretical functions of effective population size (${N}_{e}$), reference size ($N$), and genome size in terms of number of chromosomes ($k$) and the individual or average ($L$ in Morgans) size of chromosomes [5, 7, 9, 14]. Here, two such theoretical predictions of ${M}_{e}$ will be used: ${M}_{e}=2{N}_{e}Lk$ based on [8], and $\it {M}_{e}=\mathrm{2}{N}_{e}Lk/\mathrm{l}\mathrm{n}({N}_{e}L)$ based on [7].
- Based on the inverse of the variance of relationships [8]. Because ${M}_{e}$ is used to estimate the accuracy of DEBV, the variance of genomic minus pedigree relationships among all pairs of individuals in the reference population was used.
- Based on observed accuracies of GEBV and of PEBV in the reference population, ${r}_{{G}_{r}}$ and ${r}_{{A}_{r}}$, using the relationships among the accuracies derived above based on either the Fisher or the Index approach:
- Using the Fisher approach, ${\theta }_{G}$ and ${\theta }_{A}$ can be computed from the observed ${r}_{{G}_{r}}$ and ${r}_{{A}_{r}}$ using Eq. (4), with ${q}_{G}^{2}={q}_{A}^{2}=1$. Fisher information statistic ${\theta }_{D}$ can then be computed as ${\theta }_{D}={\theta }_{G}-{\theta }_{A}$ based on Eq. (5). Substituting Eq. (3) into Eq. (2) results in the following quadratic form in ${M}_{e}$: ${\theta }_{D}{M}_{e}^{2}+{\theta }_{D}M{M}_{e}-NM{h}^{2}=0$, which can be solved for ${M}_{e}$ as:
  10 $${M}_{e}=\left[{-\theta }_{D}M+\sqrt{{\theta }_{D}^{2}{M}^{2}+4{\theta }_{D}NM{h}^{2}}\right]/2{\theta }_{D}.$$
- Using the Index approach, ${r}_{{D}_{r}}^{2}$ can be computed from the observed ${r}_{{G}_{r}}$ and ${r}_{{A}_{r}}$ using Eq. (9), which can then be used to compute ${\theta }_{D}$ for a given value of ${q}_{D}^{2}$ using Eq. (6). ${M}_{e}$ can then be derived from ${\theta }_{D}$ using Eq. (2), resulting in:
  11 $${M}_{e}=N{q}_{D}^{2}{h}^{2}/{\theta }_{D}.$$

Because ${q}_{D}^{2}=M/(M+{M}_{e})$ based on Eq. (3), the solution for ${M}_{e}$ must be obtained in an iterative manner by substituting the new value of ${q}_{D}^{2}$ based on Eq. (3) back into Eq. (11) until a stable value of ${M}_{e}$ is obtained (see Appendix 1).

### Prediction of ${r}_{G}$ in the target population

The accuracy of $${\widehat{g}}_{D}$$ in the target population was modelled as the product of the accuracy of $${\widehat{g}}_{D}$$ in the reference population ($${r}_{{D}_{r}})$$ and the loss of genomic information between the reference and target population ($${p}_{rt}$$) as:

12 $${r}_{{D}_{t}}={p}_{rt}{r}_{{D}_{r}}.$$

Parameter ${p}_{rt}$ can be derived by considering that ${\widehat{g}}_{D}$ in the reference population is the sum of estimates for ${M}_{e}$ independent chromosome segments in the reference population, each with accuracy ${r}_{{D}_{r}},$ as derived above for ${\widehat{g}}_{D}.$ If an individual in the target population inherits both its paternal and maternal haplotype in a segment intact from its closest ancestors in the reference population, then that segment will maintain the same accuracy ${r}_{{D}_{r}}$ in the target population. However, if either the paternal or the maternal segment recombined between the reference and the target population, then the accuracy of that segment was assumed to be 0 for that target individual. The rationale for the latter is that, although covariates for individual SNPs are fitted in genomic prediction, with LD among SNPs in a region, predictions are implicitly based on the combination of genotypes at SNPs that an individual carries at a genomic segment, i.e. the individual’s so-called diplotype [32]. As a result, if a new diplotype is created in the target individual because the maternal and/or paternal haplotype that it received had recombined since leaving the reference population, the predictive accuracy for that segment was assumed to be lost and equal to zero. This assumption will require further validation. The probability that an individual received a recombined paternal and/or maternal segment can be derived as follows: let ${l}_{p}$ and ${l}_{m}$ be the number of generations between an individual in the target population and its closest paternal and maternal, respectively, ancestors in the reference population (= 1 if the target individuals are progeny of individuals in the reference population). Then the probability of no recombination of a segment between the reference and target population is equal to:

${p}_{rt}={(1-kL/{M}_{e})}^{{(l}_{p}{+l}_{m})},$ where $kL/{M}_{e}$ is the average size of a segment in Morgans.

Note that the derivation of the average size of independent segments based on ${M}_{e}$ assumes that segment size is entirely driven by LD. However, Habier et al. [22] showed that genomic predictions also capture co-segregation of markers and QTL within families and that co-segregation information declines more quickly over generations than LD information because it extends over larger distances than LD information. To allow for this, the average segment size was multiplied by a factor $\gamma$, resulting in:

13 $${p}_{rt}={(1-\gamma kL/{M}_{e})}^{{(l}_{p}{+l}_{m})}.$$

Here, a fixed value of $\gamma$ = 2 was used across all simulations, which was derived by calibration of the predicted against the observed accuracy of GEBV in the target population based on one set of simulations (see section on simulations). This assumption was validated based on real data but will require further validation for other situations. Note, however, that for typical values of $k$, $L$, and ${M}_{e}$, the ratio $kL/{M}_{e}$ is close to zero and in those cases, ${p}_{rt}$ is close to 1 and rather robust to the choice of $\gamma$.

Given the predicted accuracy of ${\widehat{g}}_{D}$ in the target population (${r_{{D_t}}} = \;{p_{rt}}{r_{{D_r}}},$ based on Eq. (12)), the accuracy of GEBV in the target population, ${r}_{{G}_{t}}$, can then be predicted by combining ${r}_{{D}_{t}}$ and ${r}_{{A}_{t}}$ using either the Fisher (Eqs. (5) and (6)) or the Index approach (Eq. (8)).

### Empirical accuracy of genomic and pedigree-based EBV in the reference population

As described above, important parameters for prediction of the accuracy of ${\widehat{g}}_{G}$ in the target population are the accuracies of ${\widehat{g}}_{G}$ and ${\widehat{g}}_{A}$ in the reference population, because these accuracies are required to compute the accuracy of ${\widehat{g}}_{D}$ in the reference population, as well as the value of ${M}_{e}$. Four methods can be used to estimate the accuracies of ${\widehat{g}}_{G}$ and ${\widehat{g}}_{A}$ within a population:

(i) Theoretical prediction of accuracy based on the inverse of the coefficient matrix of the mixed model equations [23], or approximations thereof [33, 34].
(ii) Theoretical prediction of accuracy based on the accuracy without selection and genetic variance under selection [27, 35].
(iii) Empirical prediction based on cross-validation, e.g. [36].
(iv) Empirical prediction using the semi-parameteric LR method of Legarra and Reverter [37].

In a population that is under selection, an important distinction must be made between the accuracy of EBV in the unselected base population and the correlation between EBV and true BV in the selected population [27, 35]. The above method (i) predicts the accuracy of EBV in the unselected population and is usually provided as auxiliary information for EBV in routine genetic evaluation of livestock populations. It predicts the accuracy of the EBV of an individual in an unselected population with the same amount of information as the individual in the population that is under selection, in terms of the number and type of phenotypes available on the individual and its relatives, including genomic information [35, 38]. However, in a population that is under selection, these accuracies overestimate the correlation between EBV and true BV because of the reduction in the genetic variance resulting from the Bulmer effect, as well as the reduced impact of pedigree information [27, 35, 39]. To account for this (method (ii)), Dekkers [35] showed that, under the infinitesimal model, the accuracy of EBV of individuals in a population under selection ($r$) is related to the accuracy of those EBV in an unselected population (from method (i) above) in the following manner:

14 $${r}^{2}=1-(1-{{r}^{\prime}}^{2}){\sigma ^{\prime}}_{G}^{2}/{\sigma }_{G}^{2},$$

where ${\sigma }_{G}^{2}$ and ${\sigma {\prime}}_{G}^{2}$ are the genetic variance in the unselected and selected population, respectively. This equation was derived using the fact that the prediction error variance of EBV is not affected by selection under the infinitesimal model [38], which also holds for genomic EBV [27, 39, 40]. This is the basis of above method (ii) for the prediction of the accuracy of EBV in a population under selection.

While estimates of the genetic variance in the unselected (base) population are generally available, a challenge for applying Eq. (14) to compute the accuracy under selection is to obtain an estimate of the genetic variance in the selected population [41, 42]. However, Eq. (14), along with the theory of selection under multivariate normality can be used to deterministically model the impact of the Bulmer effect in an ongoing breeding program on genetic parameters, accuracy, and response to selection under the infinitesimal model using pseudoBLUP, and to derive the equilibrium or asymptotic values for these parameters in a stabilized population for both single-trait and multiple-trait selection programs [27, 35, 43]. Combined with the methods developed here, these parameters enable derivation of the accuracy of GEBV in the reference population based on method (ii).

While these theoretical predictions of accuracy have proven to be useful, they are based on assumptions that may not hold in practice, especially with genomic prediction, such as those of the infinitesimal model and multi-variate normality. To overcome these limitations, empirical estimation of the accuracy of EBV using cross-validation (e.g. [36] and [37]) has gained importance over the past decade (method (iii) above). In the simulations presented in the following, leave-one-out (LOO) cross-validation [44] was used to derive the accuracy of ${\widehat{g}}_{G}$ and ${\widehat{g}}_{A}$ in the reference population. In this approach, the information of each individual is eliminated from the data one-at-a-time to estimate the GEBV of that individual using all other data. Rather than having to conduct as many genetic evaluation runs as there are individuals in the data, computationally efficient methods have been developed to obtain LOO GEBV [44, 45]. The accuracy of the LOO EBV can then be obtained based on their correlation with pre-adjusted phenotypes divided by the square root of heritability. This correlation can be computed for subsets of animals to account for the heterogeneity of the population in terms of the information that is available, e.g. by generation, sex, and/or whether they were used for breeding.

The accuracy of LOO EBV underestimates the accuracy of EBV in the reference population because it does not include own phenotype. Information from the individual’s own phenotype can be incorporated by modelling the EBV of individuals in the reference population, ${\widehat{g}}_{G}$ or ${\widehat{g}}_{A}$, as an index of the LOO EBV (${\widehat{g}}_{{i}_{LOO}}$ for $i=A$ or $G$), with LOO cross-validation accuracy ${r}_{{i}_{LOO}}$, and own phenotype, $y$ (adjusted for fixed and other random effects), as follows:

15 $${\widehat{g}}_{i}={b}_{LOO}{\widehat{g}}_{{i}_{LOO}}+{b}_{y}y.$$

Using selection index theory and after scaling by ${\sigma }_{G}^{2}$, this results in the following index weights:

16 $$\left[\begin{array}{c}{b}_{LOO}\\ {b}_{y}\end{array}\right]={\left[\begin{array}{cc}{r}_{{i}_{LOO}}^{2}& {r}_{{i}_{LOO}}^{2}\\ {r}_{{i}_{LOO}}^{2}& 1/{h}^{2}\end{array}\right]}^{-1}\left[\begin{array}{c}{r}_{{i}_{LOO}}^{2}\\ 1\end{array}\right]=\frac{1}{1-{h}^{2}{r}_{{i}_{LOO}}^{2}}\left[\begin{array}{c}1-{h}^{2}\\ {h}^{2}(1-{r}_{{i}_{LOO}}^{2})\end{array}\right],$$

and squared accuracy:

17 $${r}_{i}^{2}=({r}_{{i}_{LOO}}^{2}+{h}^{2}-2{h}^{2}{r}_{{i}_{LOO}}^{2})/(1-{h}^{2}{r}_{{i}_{LOO}}^{2}).$$

In a related approach (the above method (iv)), Reverter et al. [46]) showed that the correlation between EBV based on partial and whole data is equal to the ratio of the accuracy of EBV based on partial versus whole data. Legarra and Reverter [37] showed that this also applies to the use of pedigree versus genomic relationships, i.e.: ${r}_{{\widehat{g}}_{A},{\widehat{g}}_{G}}={r}_{{\widehat{g}}_{A}}/{r}_{{\widehat{g}}_{G}}$. Thus, if an estimate of the accuracy of ${\widehat{g}}_{A}$ in the reference population is available (${r}_{{\widehat{g}}_{A}}$), which can be based on pseudoBLUP, the accuracy of ${\widehat{g}}_{G}$ in the reference population can be derived based on the correlation of ${\widehat{g}}_{G}$ and ${\widehat{g}}_{A}$ in the reference population as:

18 $${r}_{{\widehat{g}}_{G}}={r}_{{\widehat{g}}_{A}}/{r}_{{\widehat{g}}_{A},{\widehat{g}}_{G}}.$$

### Simulations

Stochastic simulation was used to validate the developed approaches. The main purpose of the simulations was to compare alternate estimates of ${M}_{e}$ in the reference population, with the aim to identify an estimate of ${M}_{e}$ that is little affected by reference population size, heritability, and selection, such that it can be used as a population parameter. A second objective was to determine the validity of the proposed approach for prediction of the accuracy in the target population outlined in Fig. 1. Specific emphasis was on comparing and validating the Fisher and Index methods for separating information in the reference population into that contributed by pedigree versus genomic deviated from pedigree relationships and for combining those two sources of information in the target population, as well as on validating the approach used to model the loss of accuracy of genomic information between the reference and target populations. The final objective of the simulations was to evaluate the proposed methods for estimation of the accuracies of pedigree-based and genomic EBV in the reference population based on cross-validation, which are required to implement the proposed approach in practice.

Using the software XSim [47], a genome of $k$ = 9 chromosomes of $L$ = 1.5 Morgan each was simulated, using bi-allelic loci and a mutation rate of 10^–8^ at a locus per generation. Two thousand historical generations were simulated to generate stable allele frequencies and linkage disequilibrium, with random selection and mating of 250 males and 250 females per generation. After these 2000 generations, approximately 20,000 loci with a minor allele frequency (MAF) greater than 0.1 were selected, of which 1000 random loci, with equal numbers per chromosome, were selected as QTL. The remaining ~ 19,000 loci were used as genotyped markers. Additive effects of QTL were sampled from a normal distribution and the true breeding value of each individual in generation 0 of the pedigree generations was computed by summing the product of genotype (0/1/2) and effect across all QTL. The resulting breeding values in generation 0 were then centered and scaled to a standard deviation of 1. Phenotypes were simulated by adding a random normal environmental effect, resulting in a heritability of 0.2 or 0.4. Pedigree generations 1 to 10 were produced by randomly mating 10 or 40 males to 120 females, with each female producing either 12 or 24 progeny (half male/female), resulting in 1440 or 2880 phenotyped and genotyped individuals per generation. Individuals used for breeding were either randomly selected or selected based on ${\widehat{g}}_{G}$ based on GBLUP, using a genomic relationship matrix derived using methods 1 or 2 of VanRaden [48]. Pedigree-based EBV, ${\widehat{g}}_{A}$, were computed using pedigree relationships going back to generation 0. The heritability that was used to simulate the data was used for genetic evaluation.

Empirical accuracies of ${\widehat{g}}_{G}$ and ${\widehat{g}}_{A}$ in the reference or target populations were obtained as the correlation between EBV and true BV, averaged over 50 replicates. With selection, EBV and true BV were centered within generation to avoid the correlation to be affected by genetic trend.

In the presentation of results, average empirical accuracies of ${\widehat{g}}_{G}$ and ${\widehat{g}}_{A}$ across 50 replicates in the training and the target populations are presented first, followed by empirical estimates of ${M}_{e}$ derived from the average empirical accuracies in the reference population and, finally, predicted accuracies of ${\widehat{g}}_{G}$ in the target populations derived using the developed method and empirical or theoretical estimates of ${M}_{e}$ in the reference population. This was done first for reference populations of a single generation that were 1 to 5 generations separated from the target generation, in order to test the model for the loss in accuracy from the reference to the target population and to compare alternate measures of ${M}_{e}$. Results are then presented for reference populations that accumulate data across generations to predict the next generation, to more accurately mimic an ongoing breeding program. The latter was done without and with selection on GEBV. Estimates of ${M}_{e}$ in the reference population and predicted accuracies in the target populations were derived using the average empirical accuracies of ${\widehat{g}}_{G}$ and ${\widehat{g}}_{A}$ in the reference population across replicates in order to validate the proposed approaches and compare alternate measures of ${M}_{e}$ based on the Fisher or Index approach.

To evaluate the ability to estimate the accuracies of ${\widehat{g}}_{G}$ and ${\widehat{g}}_{A}$ in the reference population from available data, empirical accuracies were derived using the LOO approach based on Eq. (17), as described above for method (iii). The true heritability was used in these calculations. Empirical accuracies were also derived based on the LR approach of Eq. (18), as described above for method (iv). The correlation between ${\widehat{g}}_{A}$ and ${g}_{A}$ was used as the accuracy of ${\widehat{g}}_{A}$ in these calculations.

### Real data application

The Index method was also applied to the results of the real data genomic prediction analyses presented in Wolc et al. [18] for a multi-generational layer chicken breeding population. Results from the evaluation of the persistency of the accuracy of GEBV across generations, as presented in Fig. 4 of Wolc et al. [18], were used. In this analysis, the reference population consisted of data from 777 individuals that were genotyped for 23,356 SNPs and successive validation (target) populations consisted of the subsequent and up to the fifth generation after the reference population, one generation at a time. Parents of the first validation generation were part of the last generation of the reference population. Estimates of the average cross-validation accuracy across traits in each validation generation based on single-trait GBLUP and pedigree-based BLUP were used. For each successive validation generation, these accuracies were used to calculate the accuracy of DEBV (${r}_{{D}_{t}}$) using Eq. (9). Then, the decline in ${r}_{{D}_{t}}$ across validation generations was estimated by regressing the natural log of estimates of ${r}_{{D}_{t}}$ on the number of generations that separates the validation population from the reference population (1 to 5) based on Eq. (12). The estimate of the resulting regression coefficient was then equated to $2(1-\gamma kL/{M}_{e})$ based on Eq. (13), as the exponent ${l}_{p}+{l}_{m}$ increases by 2 at each generation, and solved for ${M}_{e}$. Here, $\gamma$ was set equal to 2 and $kL$ equal to 30, since the chromosomes that SNPs were located on summed to ~ 30 Morgan, based on [49]. An estimate of ${M}_{e}$ was also obtained from the estimate of ${r}_{D}$ in the reference data. Since a direct estimate of the latter was not available, it was estimated as the intercept of the regression equation, i.e. setting the number of generations between the validation and reference populations equal to 0. The resulting estimate of ${r}_{{D}_{r}}$ was used to obtain an alternate empirical estimate of ${M}_{e}$ by iterating on Eqs. (3) and (11). In Eq. (11), $N$ was set equal to 777, $M$ equal to 23,356, and heritability was set equal to 0.523, which was the average of the heritability estimates of the traits analyzed by Wolc et al. [18] (individual trait heritability estimates ranged from 0.25 to 0.74).

## Results

### Single generation reference populations

**Fig. 2:**
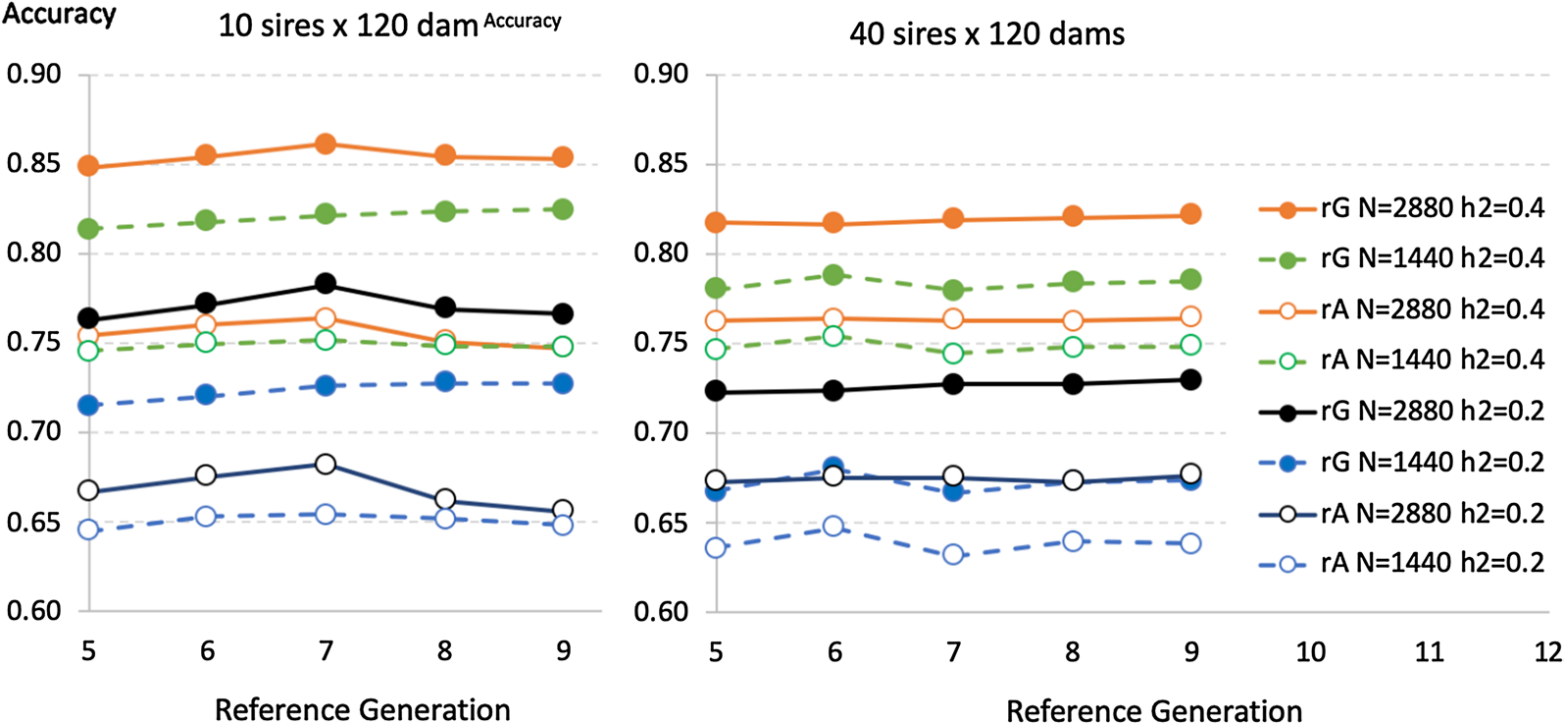
Average observed accuracy of genomic (${r}_{G}$) and pedigree-based (${r}_{A}$) predictions of breeding values in the 1-generation reference populations of generation 5 through 9. Results are based on 50 replicates for different reference data sizes ($N$), heritabilities (${h}^{2}$), and numbers of sires and dams used for breeding

To compare measures of ${M}_{e}$ in the reference population and evaluate the proposed method of predicting the loss of accuracy of ${\widehat{g}}_{G}$ from the reference to the target population, generations 5, 6, 7, 8, and 9 were used as reference population, one generation at a time, and generation 10 as the target population. Selection was at random. Size of the reference dataset was either 1440 or 2880 and heritability was 0.2 or 0.4. Figure 2 shows average empirical accuracies of ${\widehat{g}}_{G}$ and ${\widehat{g}}_{A}$ in the reference populations across 50 replicates. Average accuracies were fairly stable across the 1-generation reference populations but with a slight tendency to increase in later generations. Estimates of the accuracy of ${\widehat{g}}_{G}$ were very similar based on the use of method 1 versus method 2 of [48] to create $\mathbf{G}$ and, therefore, only results for method 2 are shown in Fig. 2.

**Fig. 3:**
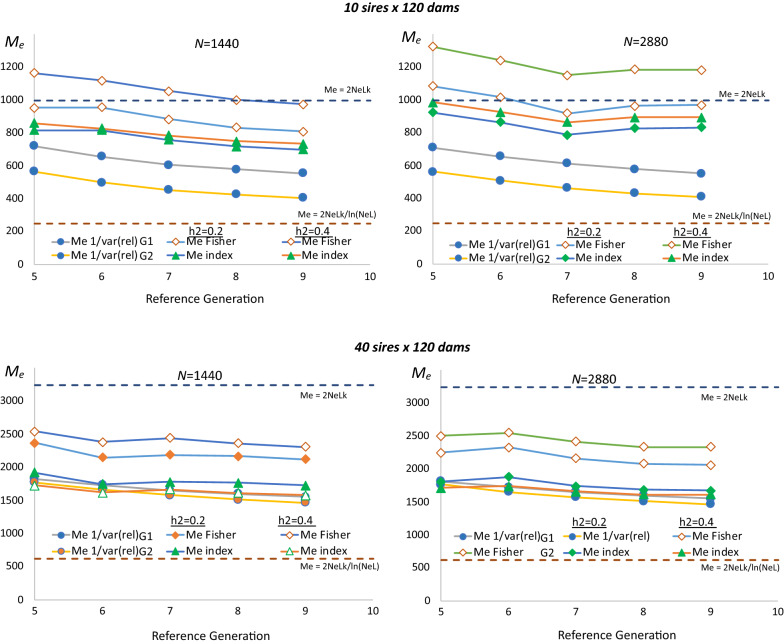
Estimates of $${M}_{e}$$ in the reference population in generations 5 through 9. Estimates are derived using different methods and for different reference data sizes ($$N$$), heritabilities ($${h}^{2}$$) and numbers of sires and dams used for breeding. $${M}_{e}$$ were estimated based on the Fisher or selection index (Index) approaches or based on the reciprocal of the variance of genomic minus pedigree-based relationships, with genomic relationships computed using method 1 (G1) or 2 (G2) of VanRaden [48]. Horizontal broken lines represent theoretical predictions of M_e_ based on the effective population size ($${N}_{e}$$ = 37 or 120) and the number ($$k$$ = 9) and size ($$L$$ = 1.5) of chromosomes based on Goddard [5] ($${M}_{e}=2{N}_{e}Lk)$$ and Goddard et al. [8] ($${M}_{e}=2{N}_{e}Lk/\mathrm{l}\mathrm{n}({N}_{e}L)$$

Empirical estimates of ${M}_{e}$ derived from the average empirical accuracies from Fig. 2 are shown in Fig. 3. Although average empirical accuracies were fairly stable over time, changes in the relative magnitude of the accuracy of ${\widehat{g}}_{G}$ versus ${\widehat{g}}_{A}$ did result in a slight decline of empirical estimates of ${M}_{e}$ over generations. The Fisher approach to derive ${M}_{e}$ (method 3a above) resulted in higher values of ${M}_{e}$ than the Index approach (method 3b above). However, estimates of ${M}_{e}$ derived based on the Index approach were less affected by reference size ($N$) and especially by ${h}^{2}$, than estimates of ${M}_{e}$ derived by using the Fisher approach, suggesting that the Index approach provides a more stable population parameter than the Fisher approach. Estimates of ${M}_{e}$ based on the inverse of the variance of relationships ($\mathbf{G}-\mathbf{A}$) were lower than corresponding estimates based on the Fisher or Index approach with 10 sires and similar to those from the Index approach with 40 sires. With 10 sires, variances of the relationships were smaller when $\mathbf{G}$ was based on method 1 of [48] compared to method 2.

**Fig. 4:**
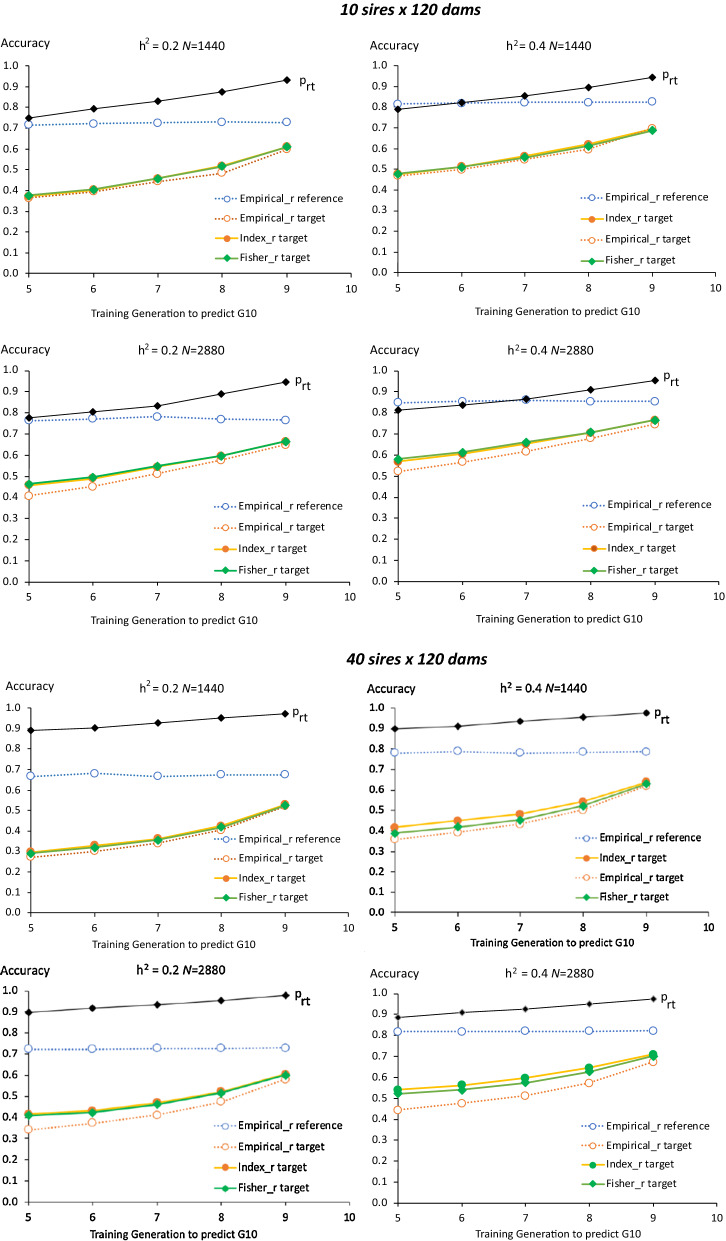
Average observed (empirical $r$) accuracies of genomic estimated breeding values in the reference (blue) and target populations (red) and predicted of accuracy of genomic estimates breeding values in the target population Results are based on 50 replicates for 1-generation reference populations of generation 5 through 9 for different reference data sizes ($N$), heritabilities (${h}^{2}$), and numbers of sires and dams used for breeding. Predicted accuracies are based on the Fisher information (green) or selection index (Index; yellow) approach. The line designated with ${p}_{rt}$ is the proportional loss from the reference to the target population in accuracy of EBV based on genomic deviated from pedigree information

Average empirical and predicted accuracies in the target population (generation 10) based on the 1-generation reference populations (generations 5 to 9) are shown in Fig. 4, along with the average empirical accuracies in the corresponding reference population across 50 replicates. Average empirical accuracies of ${\widehat{g}}_{G}$ were very similar for genomic relationships based on methods 1 or 2 of [48]. Predicted accuracies in the reference population (not shown) were identical to observed accuracies because ${M}_{e}$ was derived from the average empirical accuracies of ${\widehat{g}}_{G}$ and ${\widehat{g}}_{A}$ for each reference population. This was true for both the Fisher and the Index approach, although these two approaches resulted in different estimates of ${M}_{e}$ (Fig. 3). Note that the purpose of these simulations were not to predict the accuracy of ${\widehat{g}}_{G}$ in the reference population but to evaluate the proposed approach of separating pedigree-based and genomic information to model the accuracy of ${\widehat{g}}_{G}$ in the target population and to compare alternate measures of ${M}_{e}$ in the reference population.

Both the Fisher and the Index approach predicted the accuracy of ${\widehat{g}}_{G}$ in the target population rather well, accounting for the increase in pedigree information as the reference generation moved closer to the target generation (generation 10). However, there was some overestimation of the accuracy when the number of generations between the reference and target populations increased, especially for the larger reference size. The Fisher approach resulted in slightly higher predictions of accuracy in the target population than the Index approach because it resulted in higher estimates of ${M}_{e}$ (Fig. 3), which resulted in a smaller loss in accuracy of genomic information from the reference to the target population, based on Eq. (13).

Results in Fig. 4 were based on $\gamma$ in Eq. (13) set equal to 2, i.e. doubling the segment size relative to the estimate of ${M}_{e}$. Resulting estimates of ${p}_{rt}$ based on ${M}_{e}$ derived using the Index approach are also shown in Fig. 4 and increased almost linearly as the reference population moved closer to the target population. With $\gamma$ = 1, accuracies in the target population were overestimated when the number of generations between the reference and target populations was greater than 1, as shown in Additional file 1: Figure S1. All results presented in the remainder are based on $\gamma$ = 2.

### Multi-generation reference populations

To evaluate the developed methods within the context of an ongoing breeding program, simulations were conducted with an accumulating reference population over generations. I.e., starting with generation 0 as reference population and generation 1 as the target population, the reference population accumulated from generation 0 to 9, each time targeting the next generation. The number of genotyped and phenotyped individuals was 1440 in each generation and 10 males and 120 females were selected for breeding from the target generation. Selection was either at random or based on GEBV of individuals in the target generation prior to them being phenotyped. Results were based on the average of 50 and 30 replicates with random and GEBV selection, respectively.

**Fig. 5:**
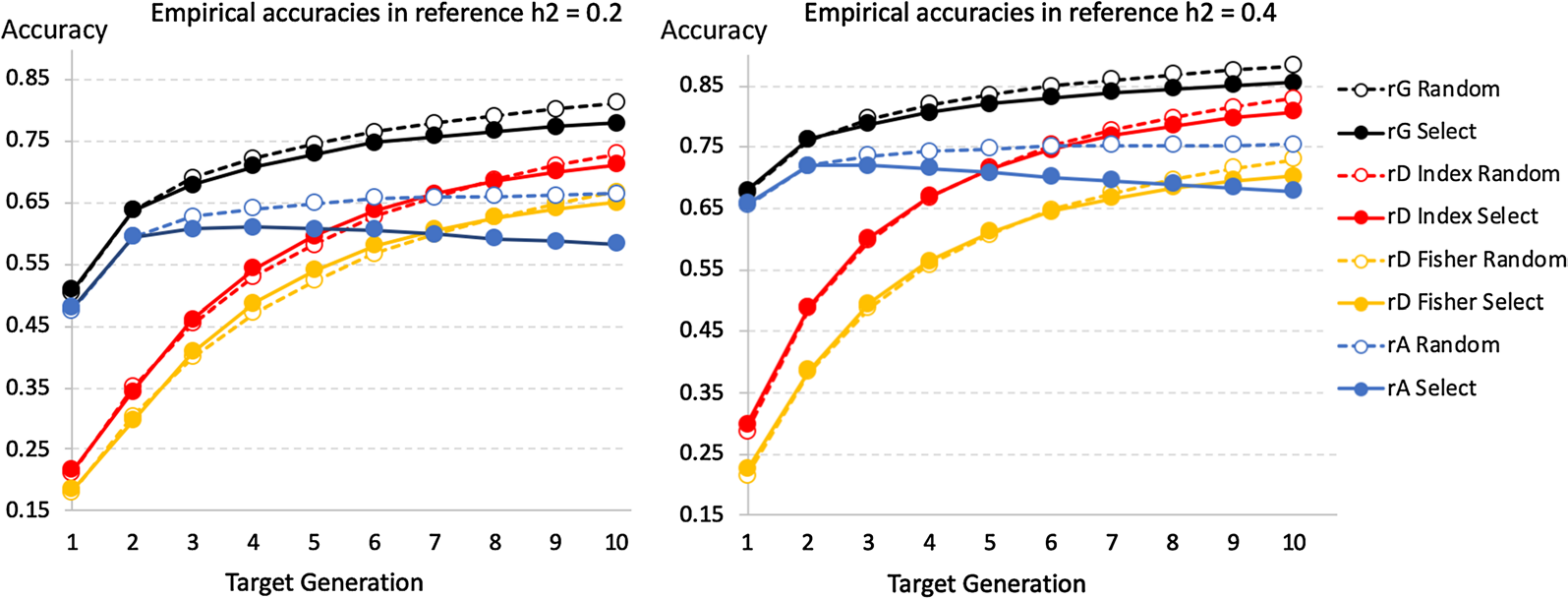
Average empirical and predicted accuracies of estimated breeding values in an accumulating reference population and breeding program with random selection (Random; broken lines) or selection on genomic estimated breeding values (Select; solid lines) of 10 males and 120 females per generation. Results are based on 50 replicates for 1-generation reference populations of generation 5 through 9 for different reference data sizes ($N$), heritabilities (${h}^{2}$), and numbers of sires and dams used for breeding. Predicted accuracies are based on the Fisher information (green) or selection index (Index; yellow) approach

Figure 5 shows the average empirical accuracies of ${\widehat{g}}_{G}$ and ${\widehat{g}}_{A}$ in the reference population, with or without selection, as well as the accuracies of ${\widehat{g}}_{D}$ (${r}_{D}$) that were derived based on these average accuracies, using the Fisher (Eq. (6)) or the Index (Eq. (9)) approach. With random selection, the accuracy of ${\widehat{g}}_{A}$ initially increased, as pedigree information accumulated, and then plateaued, as expected. With selection, the accuracy of ${\widehat{g}}_{A}$ initially increased because of the accumulation of pedigree information, similar to the case without selection, but then gradually declined, first as a result of the Bulmer effect, then because of the loss of genetic variation due to allele frequencies moving to fixation.

Derived accuracies of ${\widehat{g}}_{D}$, ${r}_{D}$, increased at a declining rate as the size of the reference population increased. Accuracies of ${\widehat{g}}_{D}$ increased faster than accuracies of ${\widehat{g}}_{G}$ and ${\widehat{g}}_{A}$ as reference size increased because genomic minus pedigree information replaces pedigree information as the size of the reference population increases and pedigree information reaches its limit. Interestingly, derived accuracies of ${\widehat{g}}_{D}$ were little affected by selection. Towards the end of the 10-generation period, accuracies of ${\widehat{g}}_{D}$ were slightly lower with selection than without selection, which is probably due to the greater loss of genetic variance beyond the Bulmer effect, which reduces the Mendelian sampling variance. The derived accuracy of ${\widehat{g}}_{D}$ was substantially lower when based on the Fisher approach compared to the Index approach. However, in both cases, ${r}_{D}$ was little affected by selection. The accuracy of ${\widehat{g}}_{G}$, which combines pedigree and genomic information, also increased at a declining rate as the size of the reference population increased. The effect of selection on reducing the accuracy was greater for ${\widehat{g}}_{G}$ than for ${\widehat{g}}_{D}$ but less than for ${\widehat{g}}_{A}$.

**Fig. 6:**
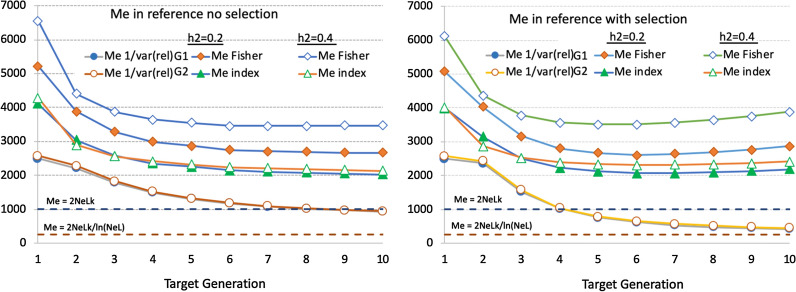
Estimates of ${M}_{e}$ in an accumulating reference population for each target generation. Estimates were derived using different methods for different heritabilities (${h}^{2}$) and with random selection or selection on genomic estimated breeding values of 10 males and 120 females from the target generation for breeding. ${M}_{e}$ were derived based on the Fisher information or selection index (Index) approaches or based on the reciprocal of the variance of genomic minus pedigree-based relationships, with genomic relationships computed using method 1 (G1) or 2 (G2) of VanRaden [48]. Horizontal broken lines represent theoretical predictions of M_e_ based on the effective population size (${N}_{e}$ = 37 or 120) and the number ($k$ = 9) and size ($L$ = 1.5) of chromosomes based on Goddard [5] (${M}_{e}=2{N}_{e}Lk$) and Goddard et al. [8] (${M}_{e}=2{N}_{e}Lk/\mathrm{l}\mathrm{n}({N}_{e}L)$)

Empirical estimates of ${M}_{e}$ derived from the average empirical accuracies from Fig. 5 are shown in Fig. 6. Estimates of ${M}_{e}$ initially decreased because the population switched from ${N}_{e}$ = 500 to 37. Estimates of ${M}_{e}$ derived using the Fisher approach were larger and were affected more by heritability than ${M}_{e}$ derived using the Index approach, again indicating that the Index approach results in a more stable estimate of ${M}_{e}$ for a population. Estimates of ${M}_{e}$ derived using either approach were not much affected by selection, although, with selection, ${M}_{e}$ based on the Fisher approach tended to increase in later generations, while ${M}_{e}$ based on the Index approach tended to plateau. Estimates of ${M}_{e}$ based on the inverse of the variance of relationships substantially underestimated ${M}_{e}$ and were substantially lower with than without selection. They were similar for relationships based on VanRaden methods 1 and 2 [48]. Theoretical estimates of ${M}_{e}$ based on effective population size also substantially underestimated ${M}_{e}$.

**Fig. 7:**
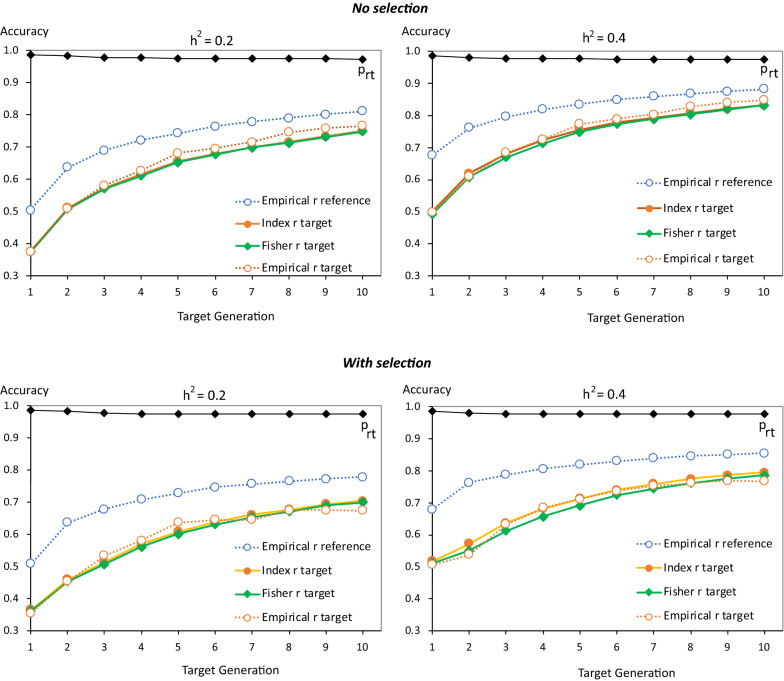
Average (50 replicates without selection, 30 with selection) empirical (broken lines) and predicted (solid lines) accuracy of genomic estimated breeding values in each target generation based on an accumulating reference population. Empirical results are based on 50 replicates with random selection and 30 replication with selection on GEBV. Results are shown for different heritabilities (${h}^{2}$), and with random selection or selection on genomic estimated breeding values of 10 males and 120 females in each target generation. Predicted accuracies were based on the Fisher (green) or selection index (Index; yellow) approach. Average empirical accuracies in the reference populations are shown also (blue). The line designated with ${p}_{rt}$ is the proportional loss from the reference to the target population in accuracy of EBV based on genomic deviated from pedigree information

Average empirical and predicted accuracies in each target generation based on the accumulating reference populations are shown in Fig. 7, along with the observed accuracy of the corresponding reference populations and estimates of ${p}_{rt}$. Again, empirical accuracies were very similar for genomic relationships based on VanRaden methods 1 and 2 of [48] for both the reference and target populations. Predicted accuracies in the reference population that were based on either the Fisher or the Index approach were again identical to empirical accuracies because estimates of ${M}_{e}$ were derived from the average empirical accuracies of ${\widehat{g}}_{G}$ and ${\widehat{g}}_{A}$ for the reference population. Both the Fisher and the Index approach correctly predicted accuracy in the target population over time, both with and without selection. Although the Fisher and Index approaches resulted in different estimates of ${M}_{e}$, they resulted in nearly identical predictions of accuracy in target population. Estimates of ${p}_{rt}$ were greater than 0.97 in all cases, because individuals in the target population were progeny of individuals in the reference population. Thus, the main reason for the drop in accuracy of ${\widehat{g}}_{G}$ from the reference to the target population was the contribution of pedigree information.

### Application to real data

**Fig. 8:**
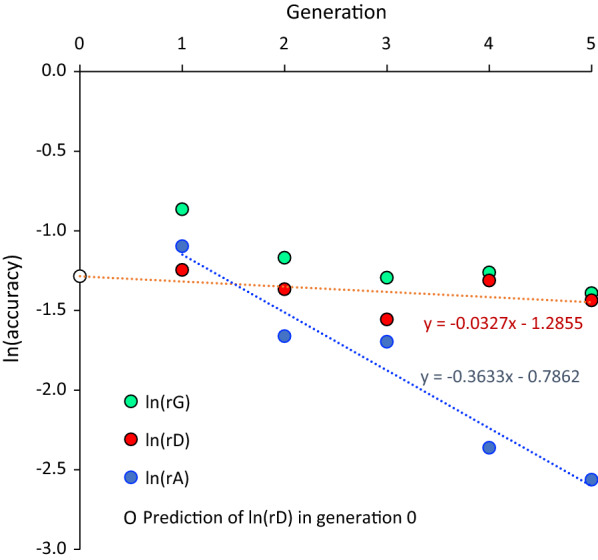
Cross-validation accuracies of genomic (${r}_{G}$) and pedigree-based (${r}_{A}$) EBV in a real chicken population and the derived accuracy of EBV based on genomic deviated from pedigree relationships (${r}_{D}$). Shown are the natural log of the accuracies of GEBV, PEBV, and DEBV in validation populations that were 1 to 5 generations removed from the reference population. The broken lines show the linear regression lines for ${r}_{D}$ (red) and ${r}_{A}$ (blue). The open circle at generation 0 is the prediction of ${r}_{D}$ based on the regression line

Figure 8 shows the natural log of the cross-validation accuracies of GEBV and PEBV in the validation populations that were 1 to 5 generations removed from the reference population, as well as the natural log of the derived ${r}_{{D}_{t}}$ for each validation generation. Declines in the log of ${r}_{{A}_{t}}$ and of ${r}_{{D}_{t}}$ by generation were approximately linear, with estimates of regression coefficients equal to − 0.3663 and − 0.0327, respectively. Note that the former corresponds to a decline of ${r}_{{A}_{t}}$ by a factor e^− 0.3663^ = 0.70, which is as expected based on pedigree. The average decline of ${r}_{{D}_{t}}$ per generation was by a factor e^− 0.0327^ = 0.968. Based on Eq. (13), the latter can be used to obtain an estimate of ${M}_{e}$ by equating 0.968 to $2(1-2*30/{M}_{e})$, resulting in ${M}_{e}$ = 3705. Alternatively, an estimate of ${M}_{e}$ can be also obtained from an estimate of ${r}_{D}$ in the reference population, which was derived based on the intercept of the estimated regression equation as ${r}_{{D}_{r}}={e}^{-1.2855}=0.277$. Using the latter to iterate on Eqs. (3) and (11) resulted in ${M}_{e}$ = 3727, which was very close to the estimate of ${M}_{e}$ based on the decline in accuracy over validation generations, validating both the estimate of ${M}_{e}$ based on the Index approach and how the decline in accuracy of genomic information over generations was modeled.

### Estimation of the accuracy of EBV in a reference population

**Fig. 9:**
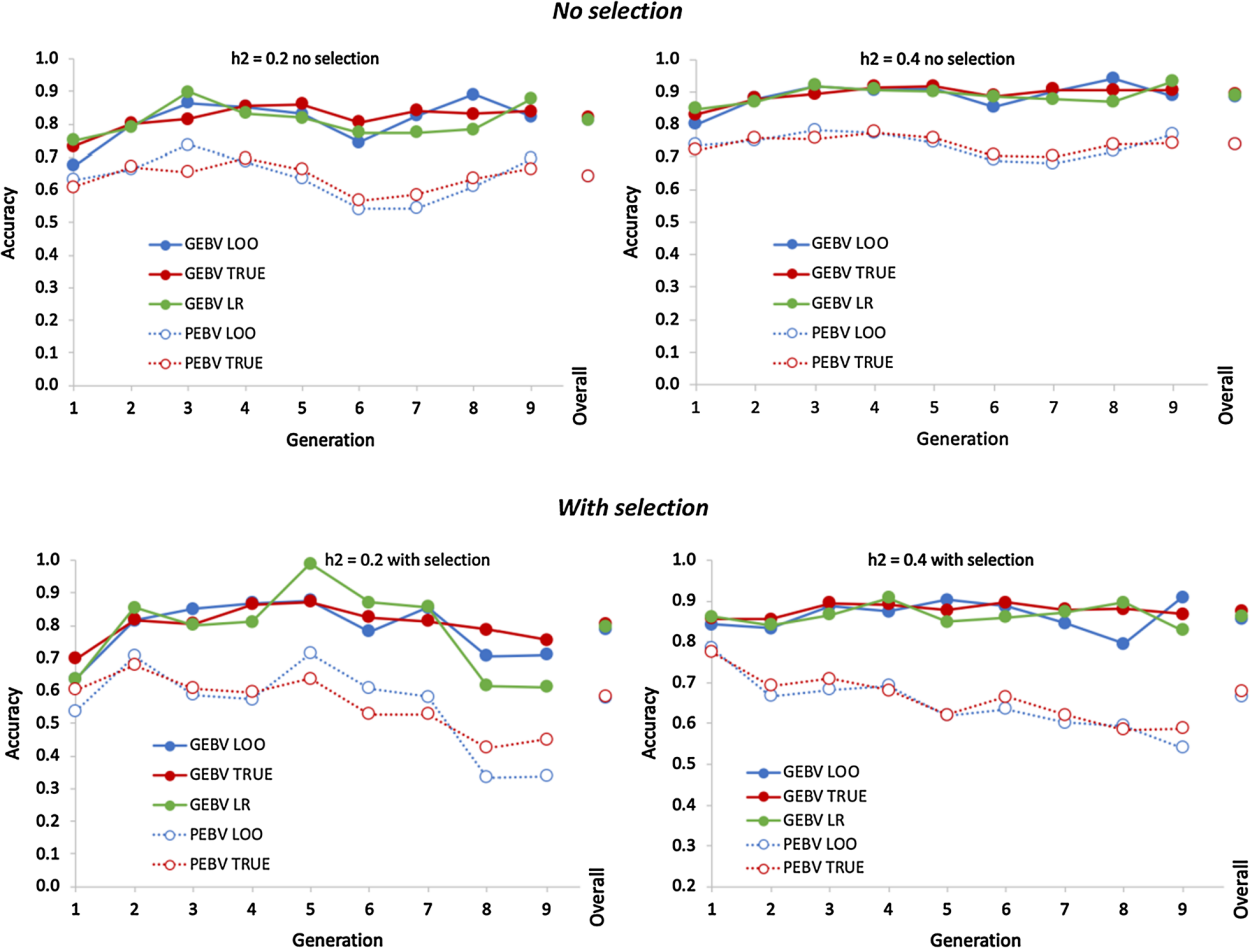
True and estimated accuracy of genomic (GEBV) and pedigree-based (PEBV) estimated breeding values in the nine-generation reference population for generation 10 (Overall) and in each of its contributing generations.Results are based on one replicate for different heritabilities (${h}^{2}$) and with random selection or selection on genomic estimated breeding values of 10 males and 120 females in each generation. Estimated accuracies were based on leave-one-out cross-validation (LOO) or based on the correlation of part-whole EBV (LR)

In the previous section, average empirical estimates of the accuracies of ${\widehat{g}}_{G}$ and ${\widehat{g}}_{A}$ in the reference population across replicates were used to derive ${M}_{e}$ in the reference population and the accuracy of ${\widehat{g}}_{D}$ in the reference population in order to compare alternate measures of ${M}_{e}$ and to validate the developed methods to predict the accuracy of ${\widehat{g}}_{G}$ in the target population. If an estimate of ${M}_{e}$ in the reference population is not available, the accuracy of ${\widehat{g}}_{G}$ and ${\widehat{g}}_{A}$ in the reference population can be estimated from available data. To demonstrate how to obtain such within-population accuracies, they were both estimated based on LOO cross-validation Eq. (17) and based on the correlation of part-whole EBV Eq. (18). Results are in Fig. 9 for one replicate of the accumulating reference population for target generation 10. For the full reference population, estimated accuracies were very close to the true accuracies, for both ${\widehat{g}}_{G}$ and ${\widehat{g}}_{A}$. For individual generations of this reference population, true and estimated accuracies fluctuated, especially when heritability was lower and with selection. For the most part, estimates of accuracy of individual generations differed from the true accuracy but not substantially and more-or-less at random. The LOO and LR approaches resulted in different estimates of accuracy for individual generations, without a consistent advantage of one approach over the other.

## Discussion

Breeding programs are complex and expensive to run and their design requires many decisions that must be optimized, including the choice of the breeding goal, the size of the breeding population, which phenotypes to measure, on which animals, and at what age, and, with the availability of high-density SNP genotyping panels, which animals to genotype, at what density, and at what age. Because of the expense and long planning horizon of breeding programs, opportunities to evaluate alternate breeding program designs by experimentation are prohibitive. Thus, instead, design and optimization of breeding programs must be based on mathematical models. One approach is to model a breeding program by stochastic simulation and many studies have used this to evaluate alternate genomic selection programs over the past decades [1, 2]. However, stochastic simulation is computationally demanding, in particular because many replicates must be run to obtain accurate estimates of the expected outcomes of breeding programs, which is the key feature of interest when comparing alternative programs. Deterministic mathematical models of breeding programs for prediction of expected responses to selection are computationally much less demanding and, as a result, allow large numbers of alternates to be evaluated and also enable optimization of breeding programs. In addition, deterministic models of breeding programs provide greater insight into the factors that affect expected outcomes. A limitation of deterministic models of breeding programs is that, by necessity, they assume the infinitesimal genetic model and multivariate normality, while stochastic simulation allows alternate genetic architectures of traits to be considered. However, for most traits of interest in animal breeding, the infinitesimal model provides good approximations to the nature of traits, especially over a limited number of generations of selection.

A key parameter for deterministic models of breeding programs is accuracy of selection. For pedigree-based breeding programs, prior to the era of genomic prediction, selection index approaches based on PseudoBLUP have been developed and used to evaluate the accuracy of PEBV [25, 26]. PseudoBLUP selection index methods were extended to incorporate information from individual genetic markers and genomic predictions [3], assuming the accuracy of such information, in terms of its correlation with the true breeding value of selection candidates, is known. Although multiple formulae and concepts to predict the accuracy of GEBV have been developed, these methods have not been able to approximate the accuracy of GEBV obtained with real data [10, 16, 17].

The method developed here is the first to explicitly account for the contribution of pedigree versus genomic information to GEBV of selection candidates, as well as the distance of selection candidates from the reference data. The resulting method enables the modeling and comparison of breeding programs that differ in the size, scope, and design of their phenotyping and genotyping programs. Ultimately, this will enable optimization of breeding programs with genomic data, including who should be genotyped and phenotyped for which traits and when, as well as optimization of multiple-stage selection programs.

The method for prediction of the accuracy of GEBV in the target population that was developed here is based on three concepts: (i) the contribution of pedigree relationships versus genomic deviations from pedigree relationships to GEBV, (ii) prediction of the accuracy of ${\widehat{g}}_{D}$ based on genomic relationships deviated from pedigree relationships in the reference population, and (iii) erosion of this genomic information between the reference and target populations. Methods to model these three concepts were developed and, using simulation, they were shown to result in accurate estimates of the accuracy of GEBV in selection candidates, both with and without selection. Aspects of the methods were also validated using real data. However, full validation of the developed method was not possible, as it requires an estimate of the accuracy in the training data, which is typically not provided.

### Modeling the contribution of pedigree information to GEBV

While it is well-known that GEBV capture pedigree information [2], most methods to predict the accuracy of GEBV have not explicitly modeled this. In fact, the original formula for prediction of the accuracy of GEBV assumed a reference population of unrelated individuals [4, 5]. Separating out pedigree-based information from GEBV in the reference population and adding it back into the GEBV for the target population was able to accurately model the decline in accuracy of GEBV as the target population was more generations separated from the reference population. This was demonstrated using both simulated and real data. Differentiating between pedigree-based and genomic information also enabled the differential rate of erosion of pedigree-based information versus genomic information to be modelled, which was recognized to be important by Habier et al. [2].

Two approaches were used to quantify the contribution of pedigree versus genomic information to GEBV: the Fisher information approach and the selection index approach. Note that both these approaches are different from the GBLUP model that was proposed by Legarra and Ducrocq [24], which simultaneously fits pedigree and genomic minus pedigree components of breeding values, such that the GEBV is the sum of the resulting predictions, ${\widehat{g}}_{A}$ and ${\widehat{g}}_{D}$: ${\widehat{g}}_{G}={\widehat{g}}_{A}+{\widehat{g}}_{D}$. The latter equation does not hold for the Fisher and Index approaches that were used here to differentiate pedigree and genomic contributions to GEBV, as both these approaches assume that the estimates ${\widehat{g}}_{A}$ and ${\widehat{g}}_{D}$ are obtained in separate evaluations rather than simultaneously, akin to single- versus multiple-trait evaluation. Both the Fisher and the Index approaches assume that the information that is used to estimate ${\widehat{g}}_{D}$ is independent of the information that is used to estimate ${\widehat{g}}_{A}$. Although both estimates are based on the same phenotypic data, independence of the information used for these two EBV is based on the use of pedigree relationships to estimate ${\widehat{g}}_{A}$, while deviations of genomic from pedigree relationships are used to estimate ${\widehat{g}}_{D}$. The assumption of independence of prediction errors of ${\widehat{g}}_{A}$ and ${\widehat{g}}_{D}$ is motivated by the independence of pedigree relationships, which contribute to ${\widehat{g}}_{A}$, from genomic minus pedigree relationships, which contribute to ${\widehat{g}}_{D}$. Although a proof of this independence was not derived, results show that any violation of this assumption appears to have limited effects on results.

To further investigate the relationships of ${\widehat{g}}_{A}$ from a pedigree-based analysis with ${\widehat{g}}_{A}^{*}$ and ${\widehat{g}}_{D}$ from the Legarra and Ducrocq [24] model, the latter were computed for a number of simulation replicates. As shown in [24], ${\widehat{g}}_{A}^{*}$ can be computed from the vector of GEBV, ${\widehat{\mathbf{g}}}_{\boldsymbol{G}}$, as: ${\widehat{\mathbf{g}}}_{A}^{\boldsymbol{*}}=\mathbf{A}{\mathbf{G}}^{-1}{\widehat{\mathbf{g}}}_{G}$, where $\mathbf{A}$ and $\mathbf{G}$ are the pedigree-based and genomic relationship matrices, respectively. Then, ${\widehat{\mathbf{g}}}_{D}$ can be computed as: ${\widehat{\mathbf{g}}}_{D}$= ${\widehat{\mathbf{g}}}_{G}$ − ${\widehat{\mathbf{g}}}_{A}^{\boldsymbol{*}}$. Results showed that ${\widehat{g}}_{A}^{*}$ and ${\widehat{g}}_{D}$ were highly negatively correlated (up to − 0.8), as expected, because their sum is equal to ${\widehat{g}}_{G}$. This high negative correlation also reflects the instability of the predictions ${\widehat{g}}_{A}^{*}$ and ${\widehat{g}}_{D}$ that are obtained from this model, although ${\widehat{g}}_{G}$ may be quite accurate. In contrast, ${\widehat{g}}_{A}$ from the pedigree-based analysis and ${\widehat{g}}_{D}$ from the Legarra and Ducrocq [24] model had very low correlations, reflecting the near independence of their prediction errors.

### ${\boldsymbol{M}}_{\boldsymbol{e}}$ in the reference population

A key parameter in deterministic methods to predict the accuracy of GEBV is the effective number of chromosome segments, ${M}_{e}$, as defined by [12] and [5]. While the original methods that used this concept referred to ${M}_{e}$ as a property of the reference data, related to the number of effects that need to be estimated, recent methods have defined ${M}_{e}$ to be between the reference and target population [8, 11]. Our results demonstrate that ${M}_{e}$ in the reference population is the key parameter that determines both the accuracy of genomic information in the reference population and the loss in accuracy of genomic information from the reference to the target population.

The relationship between ${M}_{e}$ and accuracy of genomic information in the reference population was used here to identify a measure of ${M}_{e}$ which, similar to effective population size, ${N}_{e}$, can be viewed as an inherent parameter of the population and its history in terms of family structure, rather than of the data that it generates. Derivation of ${M}_{e}$ from the accuracy of information captured by genomic minus pedigree relationships in the reference data based on the Index method provided such a measure, as it was not much affected either by reference data size and trait heritability, or by selection. This was less the case when partitioning accuracies using the Fisher approach, which was used by van den Berg et al. [17]. The importance of this result is that ${M}_{e}$ does not have to be derived separately for each trait in a population, at least when GEBV are based on the GBLUP method; van den Berg et al. [17] showed that ${M}_{e}$ for a trait tends to be smaller when variable selection methods (Bayes R) are used for genomic evaluation, depending on the number of QTL that affect the trait. This implies that ${M}_{e}$ may need to be estimated separately for each trait, if variable selection methods are used for genomic prediction. However, the number of QTL does not affect the accuracy and, therefore, ${M}_{e}$ for GBLUP [17].

In this paper, several approaches are described to estimate ${M}_{e}$ in the reference population, including deterministic estimates based on effective population size, empirical estimates based on the reciprocal of the variance of genomic deviated from pedigree relationships in the reference population, and empirical estimates derived from the accuracies of ${\widehat{g}}_{A}$ and ${\widehat{g}}_{G}$ in the reference population based on the Fisher or the Index approach. Resulting estimates of ${M}_{e}$ differed greatly between methods. Limitations of deterministic estimates of ${M}_{e}$ based on ${N}_{e}$ have been addressed in the literature [10, 16, 17] and also here, they provided poor estimates. Estimates of ${M}_{e}$ based on the reciprocal of the variance of genomic minus pedigree relationships were also found to be lower than expected, which was also observed by van den Berg et al. [17], especially when the number of sires was much smaller than the number of dams. Further work is needed to investigate the relationship of ${M}_{e}$ with the variance of relationships with a hierarchical family structure. The variance of relationships was also substantially affected by selection and continued to increase over generations, leading to a continuous decline in ${M}_{e}$ (Fig. 6). Thus, the variance of relationships does not provide an appropriate means of estimating ${M}_{e}$ in a population, as was also concluded by van den Berg et al. [17].

To evaluate empirical measures of ${M}_{e}$ based on observed accuracies of ${\widehat{g}}_{A}$ and ${\widehat{g}}_{G}$, using the Fisher or Index approach, ${M}_{e}$ was derived separately for each reference dataset. As a result, predicted accuracies of ${\widehat{g}}_{G}$ in the reference population were identical to the observed accuracies for all reference datasets for both the Fisher and Index approach. However, the purpose of these simulations was to evaluate the resulting measures of ${M}_{e}$ and how they depended on heritability, reference data size, and selection, as well as how they changed over generations. For this purpose, historical effective population size was also chosen to be substantially different from the current population size, as might be the case when a selection program is implemented in a previously unselected population. As expected, this resulted in substantial changes in estimates of ${M}_{e}$ in the initial generations (Fig. 6) but, importantly, ${M}_{e}$ tended to stabilize after a number of generations, in particular ${M}_{e}$ derived using the Index approach (Figs. 3 and 6). Thus, the Index approach can be recommended for estimation of ${M}_{e}$ as a population parameter. In practice, this implies that an estimate of ${M}_{e}$ for a population can be obtained from estimates of the accuracy of ${\widehat{g}}_{A}$ and ${\widehat{g}}_{G}$ in a reference population for one trait and then applied across traits. Alternatively, ${M}_{e}$ could be estimated separately for multiple traits and then averaged.

If empirical accuracies of ${\widehat{g}}_{A}$ and ${\widehat{g}}_{G}$ in the reference population are available, knowledge of ${M}_{e}$ is not required for derivation of the empirical accuracy contributed by genomic relationships deviated from pedigree relationships in the reference population when using the Index approach (Eq. (10)), nor is it required for combining the accuracies of ${\widehat{g}}_{A}$ and ${\widehat{g}}_{D}$ in the target population into the accuracy of ${\widehat{g}}_{G}$ in the target population. In the Index approach, ${M}_{e}$ only enters into computing the loss of the accuracy of genomic information from the reference to the target population. When using the Fisher approach, ${M}_{e}$ does enter into the partitioning of the accuracy of ${\widehat{g}}_{G}$ into the accuracies of ${\widehat{g}}_{A}$ and ${\widehat{g}}_{D}$ but only through the proportion of genetic variance that is captured by markers, i.e. ${q}^{2}$. Note that, if the number of markers is large, ${q}^{2}$ is approximately 1 and, then, ${M}_{e}$ also only enters into computing the loss of accuracy of genomic information from the reference to the target population for the Fisher approach. However, if the accuracy of GEBV in the reference population is not known, then an estimate of ${M}_{e}$ is needed to derive the accuracy of ${\widehat{g}}_{D}$ in the reference population, based on Eq. (6).

### Loss of accuracy from the reference to the target population

In the literature, several approaches have been used to model the loss of accuracy of GEBV from the reference to the target population. Habier et al. [2] modeled the erosion of the accuracy of the genomic component of GEBV over generations based on the probability of no recombination between the markers and QTL. For prediction across populations, Wientjes et al. [21] modeled the loss of accuracy of GEBV from one population to the next based on the consistency of marker-QTL LD between the reference and the target populations. They quantified this loss by the ability to predict QTL genotypes in the target population based on predictions of QTL genotypes from marker genotypes in the reference population. Karaman et al. [50] showed that the accuracy of GEBV in the target population is limited by the estimability of marker genotype combinations that are present in the target population based on genotype combinations that are present in the reference data.

The approach developed here to predict the loss of accuracy of genomic information from the reference to the target population was based on the probability that both the maternal and the paternal haplotype that a target individual received for a genome segment were inherited from the reference population without recombination, following Habier et al. [2]. If both haplotypes are inherited without recombination, then the accuracy of the estimate of that segment in the target individual is expected to be the same as the accuracy of that segment in the reference population. If one or both haplotypes are inherited with recombination, then that segment will be novel and its estimate based on the reference population was assumed to be zero. The probability of no recombination was based on the number of generations between the target individual and its closest ancestors in the reference population and the average size of each segment. The former was based on Clark et al. [19], who showed that the accuracy of GEBV was driven by the maximum relationship of the target individual with individuals in the reference population. Further research may be needed to validate this assumption, especially for multi-generational and heterogeneous data; in those cases, some weighted average of the distance of the target to individuals in the reference population may be more appropriate.

The average size of segments was initially based on ${M}_{e}$ in the reference population and genome size in Morgans. However, this resulted in underestimation of the loss of accuracy from the reference to the target population (see Additional file 1: Figure S1). This underestimation is likely because ${M}_{e}$ is based on LD in the population and ignores that part of the contribution of genomics to the accuracy of GEBV is through co-segregation between markers and QTL, as demonstrated by Habier et al. [22]. Contributions from co-segregation extend over longer genomic distances than contributions from LD [22]. Doubling the size of independent segments, which was based on correctly predicting the loss of accuracy as the number of generations between the reference and target populations increased in the simulated base scenario (comparing Fig. 4 and Additional file 1: Figure S1), was found to improve predictions of the loss of accuracy across all scenarios investigated here. However, this adjustment will require further validation and theoretical development. Ideally, contributions of genomic over pedigree information to GEBV are further separated into contributions of co-segregation versus LD, such that the decay of their information over generations can be modelled separately. In addition, the loss of accuracy should be affected by the distribution of the effect of a QTL across its neighboring markers and their distance from the QTL. This distribution will depend on the multi-locus LD of the QTL with its neighboring markers, the size of the reference population, and whether the statistical model prioritizes nearby markers. For example, markers that capture effects of a QTL are expected to be closer to the QTL when using variable selection models such as Bayes-B, compared to GBLUP [2]. Therefore, Bayes-B is expected to result in a smaller loss of accuracy of GEBV from the reference to the target population than GBLUP. However, if the size of the reference population is large, only markers very close to the QTL are expected to capture most of the effects of the QTL for both Bayes-B and GBLUP. In that case, loss of accuracy is expected to be small for both methods.

The method to model the decline in accuracy of genomic information over generations was validated using observed accuracies in the reference and validation data from a layer chicken population. Based on these accuracies, which were averaged over traits to reduce variability, ${M}_{e}$ was estimated based on the decline in accuracy of genomic information over generations, and based on the observed accuracy of genomic information in the training data. These two estimates of ${M}_{e}$ showed very good agreement, which demonstrates that, at least for this example, the proposed approach to estimate ${M}_{e}$ based on observed accuracies in the reference data and its use to model the decline in accuracy of genomic information over generations holds, including the choice of doubling the size of segments ($\gamma$ = 2). However, further research is needed to determine whether this holds for other cases. In practice, if a multi-generation reference population is available, as in the real data used here, reference-training scenarios with increasing numbers of generations between the reference and training data can be generated, as in [18], and used to calibrate $\gamma$.

Although some of the assumptions of the method used here to model the loss of accuracy of genomic information from the reference to the target populations may be violated in practice, it should be noted that this loss of accuracy is expected to be small when the target individuals are progeny of individuals in the reference populations. Thus, in that case, which will be typical for most livestock breeding programs, results will be rather insensitive to the value of ${M}_{e}$ used to estimate that loss, as well as the choice of $\gamma$. Instead, most of the loss of accuracy of GEBV in those cases results from the erosion of pedigree information and, therefore, depends on the contribution of pedigree information, which appears to be accurately modeled in the proposed approach.

The loss in accuracy of DEBV from the reference to the target population by recombination could also explain the inflation of GEBV that is often observed for genomic predictions, as quantified by the regression of phenotype on GEBV of validation animals being less than 1 [37]. Based on BLUP theory [38], a decline in accuracy should result in a corresponding decline in the variance of DEBV among selection candidates compared to the reference population. However, considering that the GEBV of a selection candidate is based on the sum of the product of the candidate’s SNP genotype codes and SNP effect estimates from the reference population, the variance of DEBV of selection candidates is not expected to be lower than that of DEBV in the reference population, resulting in inflation of the GEBV of selection candidates.

### Accuracy of genomic information in the reference population

Key elements for predicting the accuracy of GEBV in the target population using the method developed here are the accuracies of PEBV and GEBV in the reference population. In the simulations, accuracies in the reference population were based on the correlation between true and estimated BV across all individuals in the reference population, with correction for genetic trend in the case of selection. Note that the goal here is to obtain the population-based accuracy of GEBV, rather than the accuracy of the GEBV of individual animals. As defined by Bijma [27], the accuracy of the EBV of an individual is defined based on the prediction error variance of the individual’s EBV, i.e. over repeated sampling, and can be derived from the inverse of the coefficient matrix of the mixed model equations [23]. However, what is relevant for prediction of response to selection, is the population accuracy, which is defined as the correlation between EBV and true BV across the population [27]. In a population that is under selection, it is important for both the individual and population accuracies to be adjusted for the effect of selection, as described by Dekkers [35] and Bijma [27], in order not to overestimate accuracy. Note also that the goal here is to estimate the accuracy of within-sample prediction, i.e. the accuracy within the reference data, rather than the accuracy of predictions of breeding values or phenotypes of individuals that are not in the reference data.

In a reference population that consists of multiple generations, accuracies of EBV were found to differ between generations (Fig. 9), especially accuracies of PEBV. In addition, within a generation, accuracies differed substantially between animals that were used as parents versus those that were not. To derive the contribution of genomic information deviated from pedigree information to GEBV, however, the pooled accuracy across generations was used and found to lead to good predictions of the accuracy of GEBV in the target population.

Several approaches were described to obtain an empirical estimate of the accuracy of GEBV in the reference population. A standard approach is to conduct k-fold cross-validation in the reference data [51], where the reference data is split into k subsets and the EBV for each subset are then estimated using the data from all other subsets. This results in a cross-validation EBV for each individual in the full data and the accuracy of these EBV is then obtained by dividing their correlation with observed phenotypes by the square root of heritability. A limitation of this approach is that the resulting estimate of accuracy depends highly on how the dataset is split into subsets and whether close relatives are spread across subsets. For example, the estimate of accuracy will be lower if subsets are created by k-means clustering on relationships, which minimizes relationships between subsets [51].

Leave-one-out cross-validation was proposed here as one approach to obtain an empirical estimate of the population accuracy of EBV in the reference population. Note that this approach is not recommended to obtain the accuracy of GEBV of selection candidates. Efficient methods to obtain LOO EBV have been developed [44, 45] and were recently extended to complex mixed linear models with multiple random effects and without requiring pre-adjustment of phenotypes for fixed effects by Cheng et al. [52]. This approach also allows the phenotype of a validation individual to be corrected for fixed effects estimated using the LOO data, rather than the complete data. Although further research is needed, this approach provided accurate estimates of the accuracy of EBV under simulation. However, this method requires an estimate of heritability in the reference population, both for converting the validation correlation to an accuracy of EBV and when adding information from own phenotype to the EBV. In a population that is under selection, this heritability may be difficult to obtain. One solution would be to estimate the decrease in genetic variance as a result of the Bulmer effect using an approximate deterministic model of the breeding program, e.g. following Dekkers [3].

Another approach that was explored to obtain an empirical estimate of the accuracy of GEBV in the reference population was the correlation between part-whole EBV, applied to PEBV versus GEBV, as proposed by Legarra and Reverter [37]. This approach could be used in combination with the LOO approach, e.g. by deriving the accuracy of pedigree-based LOO EBV and the correlation of these PEBV with GEBV using the full data. Alternatively, the accuracy of PEBV could be derived deterministically using a PseudoBLUP approach and divided by the correlation between PEBV and GEBV from the data to derive the accuracy of GEBV in the reference population. The best approach to derive the accuracies of PEBV and GEBV in the reference population requires further investigation.

An interesting finding from the simulations was that the accuracy of DEBV in the reference population was not affected by selection (Fig. 5). This is likely because DEBV are based on deviations of genomic from pedigree relationships, i.e. based on Mendelian sampling terms, which are not affected by the Bulmer effect. However, in the simulations, the genetic variance continued to decrease over generations because of inbreeding and, with selection, fixation of favorable QTL alleles. Whether these relationships are as expected requires further investigation.

## Conclusions

A deterministic method was developed for the prediction of the accuracy of GEBV of selection candidates within a breeding program based on the accuracy of GEBV and PEBV in the reference population and the distance of selection candidates from their closest ancestors in the reference population. The method uses the fact that GEBV are a combination of PEBV and EBV that are based on genomic relationships deviated from pedigree (DEBV). Assuming that these two EBV have independent sampling errors, the accuracy of GEBV can be partitioned into the accuracy of these respective EBV based on selection index theory or based on Fisher information theory. Loss of the accuracy of DEBV from the reference to the target population depends on the effective number of chromosome segments in the reference population (${M}_{e}$), which determines the size of independent segments whose effects are estimated in the reference population and the probability that a random segment is broken up by recombination when moving from the reference to the target population. ${M}_{e}$ in the reference population can be estimated based on the observed accuracies of GEBV and PEBV in the reference population, using either the Fisher or the Index approach. Both the Fisher and Index approach correctly predicted the accuracy of GEBV in the target population over time, both with and without selection. The Fisher and Index approaches, however, resulted in different estimates of ${M}_{e}$, with the Index approach resulting in estimates that were less affected by heritability, reference size, and selection, and which are, therefore, more appropriate as a population parameter.

### Supplementary Information

**Additional file 1: Figure S1. **Average (50 replicates) observed and predicted of accuracy of genomic breeding values in the target population based on 1-generation reference populations of generation 5 through 9 for different reference data sizes ($N$ heritabilities (${h}^{2}$), and numbers of sires and dams used for breeding, with $\gamma$ in Eq. [12] set equal to 1 rather than 2, which is used for the results presented in the paper.

## Data Availability

Simulations are based on the published software Xsim. The real data used are from a commercial source and not publicly available.

## Appendix 1

### Pseudo-code to predict the accuracy of GEBV in the target population using the selection index method

Based on variables and parameters as defined in the text.

#### A reference population is available

1. Estimate the accuracy of GEBV in the reference population, ${r_{G{}_{r^{,}}}}$by cross-validation.
2. Estimate the accuracy of PEBV in the reference population, ${r_{{A_{r^{,}}}}}$estimated by cross-validation or by Pseudo-BLUP).
3. Compute the accuracy of DEBV in the reference population based on Eq. (9):
  $${r}_{{D}_{r}}=\sqrt{\frac{{r}_{{G}_{r}}^{2}{-r}_{{A}_{r}}^{2}}{{1+r}_{{A}_{r}}^{2}({r}_{{G}_{r}}^{2}-2)}}.$$
4. Set the proportion of genetic variance captured by markers for DEBV equal to 1: ${q}_{D}^{2}$ = 1.
5. Compute ${\theta }_{{D}_{r}}$ in the reference population using Eq. (4): ${\theta }_{{D}_{r}}=\frac{{r}_{{D}_{r}}^{2}(1-{r}_{{D}_{r}}^{2}{q}_{D}^{2}{h}^{2})}{{q}_{D}^{2}-{r}_{{D}_{r}}^{2}}.$
6. Compute *M*_*e*_ in the reference population based on Eq. (11): ${M}_{e}=N{q}_{D}^{2}{h}^{2}/{\theta }_{{D}_{r}}.$
7. Update ${q}_{D}^{2}$ using Eq. (3): ${q}_{D}^{2}=M/(M+{M}_{e}).$
8. Return to step 5) until a stable value for ${M}_{e}$ is obtained.
9. Compute the accuracy of PEBV in the target population by Pseudo-BLUP.
10. Compute the loss of accuracy of DEBV from the reference to the target population using Eq. (13) as: ${{p}_{rt}=(1-\gamma kL/{M}_{e})}^{{(l}_{p}{+l}_{m})}$, with $\gamma =2$ or obtained by calibration.
11. Compute the accuracy of DEBV in the target population using Eq. (12): ${r}_{{D}_{t}}={p}_{rt}{r}_{{D}_{r}}.$
12. Compute the accuracy of GEBV in the target population using Eq. (8): ${r}_{{G}_{t}}=\sqrt{\frac{{r}_{{A}_{t}}^{2}{+r}_{{D}_{t}}^{2}-2{r}_{{A}_{t}}^{2}{r}_{{D}_{t}}^{2}}{{1-r}_{{A}_{t}}^{2}{r}_{{D}_{t}}^{2}}}.$

#### A reference population is not available

1. Obtain an estimate of ${M}_{e}$ in the reference population by other means, i.e. based on theory or based on an estimate of ${M}_{e}$ from a comparable reference population.
2. Compute ${q}_{D}^{2}$ using Eq. (3): ${q}_{D}^{2}=M/(M+{M}_{e}).$
3. Compute ${\theta }_{{D}_{r}}$ in the reference population using Eq. (4): ${\theta }_{{D}_{r}}=\frac{{r}_{{D}_{r}}^{2}(1-{r}_{{D}_{r}}^{2}{q}_{D}^{2}{h}^{2})}{{q}_{D}^{2}-{r}_{{D}_{r}}^{2}}.$
4. Compute the accuracy of DEBV in the reference population based on Eq. (6): ${r}_{{D}_{r}}=\sqrt{\left[1+{\theta }_{{D}_{r}}-\sqrt{{\left(1+{\theta }_{{D}_{r}}\right)}^{2}-4{h}^{2}{q}_{D}^{4}}{\theta }_{{D}_{r}}\right]/2{q}_{D}^{2}{h}^{2}}.$
5. Go to step 9) under the above paragraph “A reference population is available”.

## Appendix 2

### Derivation of the accuracy of GEBV using the selection index theory

Based on variables and parameters as defined in the text.

The selection index to combine ${\hat g_A}\;{and}\,{\hat g_D}$ is:

19 $${\widehat{g}}_{G}={b}_{A}{\widehat{g}}_{A}+{b}_{D}{\widehat{g}}_{D}.$$

Using standard selection index theory [30], optimal index weights can be derived as:

$$\left[\begin{array}{c}{b}_{A}\\ {b}_{D}\end{array}\right]={\left[\begin{array}{cc}var({\widehat{g}}_{A})& cov({\widehat{g}}_{A},{\widehat{g}}_{D})\\ cov({\widehat{g}}_{A},{\widehat{g}}_{D})& var({\widehat{g}}_{D})\end{array}\right]}^{-1}\left[\begin{array}{c}cov({\widehat{g}}_{A}{,g}_{G})\\ cov({\widehat{g}}_{D}{,g}_{DG})\end{array}\right]={\left[\begin{array}{cc}{r}_{A}^{2}& {r}_{{\widehat{g}}_{A},{\widehat{g}}_{D}}\\ {r}_{{\widehat{g}}_{A},{\widehat{g}}_{D}}& {r}_{D}^{2}\end{array}\right]}^{-1}\left[\begin{array}{c}{r}_{A}^{2}\\ {r}_{D}^{2}\end{array}\right]=$$

$$\frac{1}{{r}_{A}^{2}{r}_{D}^{2}-{{r}_{{\widehat{g}}_{A},{\widehat{g}}_{D}}}^{2}}\left[\begin{array}{c}{r}_{A}^{2}{r}_{D}^{2}-{{r}_{D}^{2}r}_{{\widehat{g}}_{A},{\widehat{g}}_{D}}\\ {r}_{A}^{2}{r}_{D}^{2}-{{r}_{A}^{2}r}_{{\widehat{g}}_{A},{\widehat{g}}_{D}}\end{array}\right],$$

where ${r}_{{\widehat{g}}_{A},{\widehat{g}}_{D}}$ is the correlation between ${\hat g_A}\;{\text{and }}{\hat g_D}$. If sampling errors of ${\hat g_A}\;{and}\,{\hat g_D}$ are independent, ${r}_{{\widehat{g}}_{A},{\widehat{g}}_{D}}={r}_{A}^{2}{r}_{D}^{2}$ and $$\left[\begin{array}{c}{b}_{A}\\ {b}_{D}\end{array}\right]=$$ $\frac{1}{{1-r}_{A}^{2}{r}_{D}^{2}}$ $$\left[\begin{array}{c}1-{r}_{D}^{2}\\ 1-{r}_{A}^{2}\end{array}\right].$$ The squared accuracy of the resulting index can be derived as: $${r}_{G}^{2}={\left[\begin{array}{c}{b}_{A}\\ {b}_{D}\end{array}\right]}^{\text{'}}\left[\begin{array}{c}cov({\widehat{g}}_{A}{,g}_{G})\\ cov({\widehat{g}}_{D}{,g}_{DG})\end{array}\right]=\frac{{r}_{A}^{2}{+r}_{D}^{2}-2{r}_{A}^{2}{r}_{D}^{2}}{{1-r}_{A}^{2}{r}_{D}^{2}}.$$
