# Correction to: Predicting the accuracy of genomic predictions

**DOI:** 10.1186/s12711-021-00675-6

**Published:** 2021-10-15

**Authors:** Jack C. M. Dekkers, Hailin Su, Jian Cheng

**Affiliations:** Department of Animal Science, Iowa State University, Ames, IA USA

## Correction to: Genet Sel Evol (2021) 53:55 https://doi.org/10.1186/s12711-021-00647-w

After publication of [1], several errors in references to equations were noted.

In the Methods section entitled “${M}_{e}$ for the reference population”, reference to Eqs. (6), (7), and (9) must be replaced by Eqs. (5), (6), and (8) in the sentence: “These two accuracies can then be combined to predict ${r}_{{G}_{r}}$, using either the Fisher approach (Eqs. (5) and (6)) or the Index approach (Eq. (8)).”

In the subsequent paragraph, (3)(b), reference to Eq. (6) must be replaced by Eq. (4) in the sentence: “Using the Index approach, ${r}_{{D}_{r}}^{2}$ can be computed from the observed ${r}_{{G}_{r}}$ and ${r}_{{A}_{r}}$ using Eq. (9), which can then be used to compute ${\theta }_{D}$ for a given value of ${q}_{D}^{2}$ using Eq. (4).”

Also, in Appendix 2, the off-diagonals in the matrix must be multiplied by ${r}_{A}{r}_{D}$ in:

$$\left[\begin{array}{c}{b}_{A}\\ {b}_{D}\end{array}\right]={\left[\begin{array}{cc}{r}_{A}^{2}& {r}_{{\widehat{g}}_{A},{\widehat{g}}_{D}}{r}_{A}{r}_{D}\\ {r}_{{\widehat{g}}_{A},{\widehat{g}}_{D}}{r}_{A}{r}_{D}& {r}_{D}^{2}\end{array}\right]}^{-1}\left[\begin{array}{c}{r}_{A}^{2}\\ {r}_{D}^{2}\end{array}\right]$$

which then results in:

$$= \frac{1}{{r}_{A}{r}_{D}\left(1-{{r}_{{\widehat{g}}_{A},{\widehat{g}}_{D}}}^{2}\right)}\left[\begin{array}{c}{r}_{A}{r}_{D}-{{r}_{D}^{2}r}_{{\widehat{g}}_{A},{\widehat{g}}_{D}}\\ {r}_{A}{r}_{D}-{{r}_{A}^{2}r}_{{\widehat{g}}_{A},{\widehat{g}}_{D}}\end{array}\right]$$

In the next line, the square is missing on the left-hand-side of: ${r}_{{\widehat{g}}_{A},{\widehat{g}}_{D}}^{2}={r}_{A}^{2}{r}_{D}^{2}$

In the last line, the subscript in $cov({\widehat{g}}_{D}{,g}_{DG})$ should be G instead of DG, i.e.

$${r}_{G}^{2}={\left[\begin{array}{c}{b}_{A}\\ {b}_{D}\end{array}\right]}^{^{\prime}}\left[\begin{array}{c}cov({\widehat{g}}_{A}{,g}_{G})\\ cov({\widehat{g}}_{D}{,g}_{G})\end{array}\right]$$
