# Supplementary material for: Predicting the accuracy of genomic predictions
Source: Genet Sel Evol. 2021 Jun 29;53:55. doi: 10.1186/s12711-021-00647-w (PMC8244147; doi:10.1186/s12711-021-00647-w)
Supplement: Supplementary file 1 — Additional file 1: Figure S1. Average (50 replicates) observed and predicted of accuracy of genomic breeding values in the target population based on 1-generation reference populations of generation 5 through 9 for different reference data sizes ($N$N heritabilities (${h}^{2}$h2), and numbers of sires and dams used for breeding, with $\gamma$γ in Eq. [12] set equal to 1 rather than 2, which is used for the results presented in the paper. [file 12711_2021_647_MOESM1_ESM.docx]

Accuracy

Accuracy

Accuracy

Accuracy
